# OVEX1, a novel chicken endogenous retrovirus with sex-specific and left-right asymmetrical expression in gonads

**DOI:** 10.1186/1742-4690-6-59

**Published:** 2009-06-17

**Authors:** Danièle Carré-Eusèbe, Noëlline Coudouel, Solange Magre

**Affiliations:** 1 Endocrinologie et Génétique de la Reproduction et du Développement, INSERM, U782, 32 rue des Carnets, F-92140, Clamart – France; 2 Univ. Paris-Sud, UMR-S0782, Clamart, F-92140; 3 Physiologie de l'Axe Gonadotrope, Unité de Biologie Fonctionnelle et Adaptative (BFA), Univ. PARIS 7 – CNRS, 4 rue MA Lagroua Weill-Hallé, 75205 Paris CEDEX 13 – France

## Abstract

**Background:**

In chickens, as in most birds, female gonad morphogenesis is asymmetrical. Gonads appear first rather similarly, but only the left one undergoes full differentiation and gives rise to a functional ovary. The right gonad, in which the cortex does not develop, remains restricted to the medulla and finally regresses. Opportunity was taken of this left-right asymmetry to perform a suppression subtractive hybridization screening to select for transcripts preferentially expressed in the developing left ovary as compared to the right one, and thus identify genes that are potentially involved in the process of ovarian differentiation.

**Results:**

One of these transcripts, named *Ovex1 *according to its expression profile, corresponds to an endogenous retrovirus that has not been previously characterized. It is transcribed as full-length and singly spliced mRNAs and contains three uninterrupted open reading frames coding potentially for proteins with homology to Gag and Pro-Pol retroviral polyproteins and a third protein showing only a weak similarity with Env glycoproteins. *Ovex1 *is severely degenerated; it is devoid of typical long terminal repeats and displays some evidence of recombination. An orthologous *Ovex1 *locus was identified in the genome of zebra finch, a member of a different bird order, and similar sequences were detected in turkey, guinea fowl, and duck DNA. The relationship between these sequences follows the bird phylogeny, suggesting vertical transmission of the endogenous retrovirus for more than 100 million years.

*Ovex1 *is transcribed in chicken gonads with a sex-dependent and left-right asymmetrical pattern. It is first expressed in the cortex of the left indifferent gonads of both sexes. Expression is transient in the left testis and absent in the right one. In developing ovaries, *Ovex1 *transcription increases sharply in the left cortex and is weakly detected in the medulla. After folliculogenesis, *Ovex1*-expressing cells constitute the follicular granulosa cell layer. *Ovex1 *expression highlights a striking desquamation process that leads to profound cortical remodeling associated with follicle morphogenesis.

**Conclusion:**

Evidence for a selection pressure at the protein level suggests that this endogenous retrovirus, expressed in the ovarian supporting cell lineage, might play an active role in bird ovarian physiology.

## Background

In chickens, as in most birds, gonad differentiation is characterized by left-right (L/R) asymmetry. In the female, only the left gonad becomes a functional ovary. The right one fails to fully differentiate and ultimately disappears. By contrast, both male gonads, initially asymmetrical, become functional testes. Bird sex determination is not fully understood. The heterogametic sex, with a ZW karyotype, is female and the homogametic sex, with two Z chromosomes, is male. Unlike in mammals, the initial genetic sex trigger is not clearly identified (reviewed in [1,2]).

Gonad organogenesis begins around the 4^th ^day of incubation (E4), with identification of the genital ridge, a thickening of the coelomic epithelium on the medial aspect of the mesonephros, in which primordial germ cells, migrating from the germinal crescent, are going to settle [3]. In both sexes, the gonad epithelium is characterized by the expression of the Lim homeodomain-containing protein Lhx9 [4]. The subjacent mesenchyme, which will give rise to the so-called "medulla", expresses the steroidogenic factor SF-1 (Nr5a1/Ad4BP), Wnt-4, and by day 5, anti-Müllerian hormone (AMH) [4-7]. Early L/R asymmetry is observed in both sexes. The left gonad is larger, has a thicker epithelium, usually called "cortex", and is colonized by a greater number of primordial germ cells. This asymmetry, more pronounced in females than in males, is considered an early sign of some sex differentiation, prior to other morphological changes [8]. L/R asymmetrical expression of several genes (*estrogen receptor ER-α*, *FET-1*, *Bmp7*, *R-Spondin1*) in female gonads had been reported and was related to the asymmetry of their differentiation [9-13]. However, the mechanism responsible for this asymmetry has been identified only recently [14-16]. It depends on the bicoid type homeobox gene *Pitx2 *(Pituitary homeobox 2), a general actor in early embryo L/R differentiation [17]. In both sexes, *Pitx2c *is asymmetrically expressed in the left lateral plate mesoderm, and later in the epithelium of the left coelomic cavity in the region of gonad formation. This factor is sufficient to induce the differentiation of gonads along a "left pathway". The action of Pitx2c results from its effect on retinoic acid (RA) signaling. A balance between RA-synthesizing enzymes (RALDH2) and RA-metabolizing enzymes (CYP26) controls RA levels. In both sexes, *Pitx2c *is expressed in the cortex of the left gonad. Pitx2c repression of RALDH2 synthesis prevents RA formation. In the cortex of the right gonad, where *Pitx2c *is not expressed, RA is formed and, as CYP26 is absent, the RA cascade is activated. RA suppresses transcription of *ER-α *and of *SF-1*, a factor required for the expression of the cell cycle mediator *cyclin D1*, thus limiting cortical cell proliferation. Conversely, in the left cortex, where RA is absent, *ER-α *and *SF-1 *are expressed, and cyclin D1-stimulated cell proliferation is activated. In the medulla of both gonads, RA is degraded by Cyp26A1, which allows some *ER-α *expression.

After 6.5 days of incubation, ovaries and testes can be distinguished histologically by the differentiation in the internal region of the male gonads of testicular cords delimited by a basement membrane, enclosing germ cells and supporting Sertoli cells. These somatic cells express high levels of SOX9, DMRT1 and AMH [7,18-22].

At the same time, aromatase (P450 arom), the enzyme converting androgens to estrogens, begins to be present in the female gonads but is not expressed in the male ones [9,10,23,24]. Estrogens are essential for ovarian differentiation [25-27]. The medulla is rather similar in both female gonads. It expresses the estrogen receptor ER-α, aromatase and FoxL2, a forkhead transcription factor [28]. On the other hand, the expression of ER-α in the left cortex and its absence in the right one leads to an asymmetry in the estrogen-mediated differentiation of the gonads. Primordial germ cells and somatic cells multiply in the left ovarian cortex. Germ cells enter meiosis at E15.5 [29]. Folliculogenesis occurs after hatching at E21, with the progressive formation of follicles constituted of an oocyte surrounded by a layer of somatic granulosa cells. In contrast, the development of the right gonad is limited. The surface epithelium devoid of ER-α is not stimulated by estrogens and does not proliferate. The few germ cells do not enter meiosis, and the gonad becomes vestigial after hatching. The female right gonad does not have the physiological potential to form a functional ovary and even, in the case of left ovary castration, differentiates into a testis [30]. However, early *Pitx2c *ectopic expression is able to overcome the degenerative fate of the right gonad and to direct it toward an ovarian differentiation pathway [14].

With the purpose of finding yet unknown factors involved in the early steps of ovarian cortex differentiation, we took advantage of the differential fate of the two female gonads in chicken. Suppression subtractive hybridization screening (SSH) was used to select transcripts expressed in the left differentiating ovary and underrepresented in the right gonad. In the course of this study, we identified a new endogenous retroviral element that we named *Ovex1*, whose specific expression in gonads is characterized by a sexual dimorphism and a L/R asymmetry.

More than 30,000 endogenous retroviruses or ERVs, representing 2.9% of the DNA, are present in the chicken genome [31,32]. They are remnants of ancestral retroviruses that have gained access to the germ line of a host, leading to vertical transmission of the integrated provirus to the offspring in a Mendelian fashion. They are characterized by the presence of two long terminal repeats (LTRs), flanking sequences coding for structural and enzymatic viral proteins. The genomic organization of simple retroviruses is 5'LTR-*Gag-Pro-Pol-Env*-3'LTR. Viral protein expression is controlled by the promoter and enhancer elements located in the 5' LTR. The polyprotein Gag (group specific antigen) is a structural component of the virus particle. Pro encodes an aspartyl protease required for processing of the Gag precursor. The polyprotein Pol contains domains for reverse transcriptase (RT), RNase H, and integrase (IN). The envelope protein (Env) is composed of two domains, a surface region (SU) and a transmembrane domain (TM). After their initial integration, ERVs can copy themselves to different locations within the genome, giving rise over long periods of time to a family of related ERV elements, most of them inactivated by mutations (for a review, see [33]). Retroviruses are divided into three major classes. Class I contains elements related to gammaretroviruses (such as Moloney murine leukemia virus, MMLV) and epsilonretroviruses (as Walleye dermal sarcoma virus, WDSV). Class II elements are related to alpharetroviruses (as the Avian leukosis virus, ALV), betaretroviruses (as Mouse mammary tumor virus, MMTV), deltaretroviruses (as Human T-lymphotropic virus, HTLV) and lentiviruses (as Human immunodeficiency virus, HIV). Class III contains elements related to spumaviruses (as Human foamy virus, HFV) and ERV-L elements [34]. Some fish viruses, as the Snakehead fish retrovirus, SnRV, have an intermediate position and are said to be epsilon-like [35]. Endogenous retroviruses of the chicken remain incompletely described in spite of intensive studies. The most studied chicken ERVs are class II elements specific for *Gallus *species. The existence of retroviral sequences, related to human ERVs and representative of the other classes of retroviruses has been reported [31,32,35,36], but little is known about these elements.

Chicken *Ovex1 *contains three long open reading frames (ORF). The first two consecutive ORFs are similar to *Gag *and *Pro-Pol *retroviral sequences. The third ORF in a different frame is possibly related to *Env*. A sequence orthologous to chicken *Ovex1 *was found in the genome of zebra finch. We also detected the presence of similar *Gag *and *Pol *sequences in the DNA of three other domestic birds. Expression of *Ovex1 *was analyzed by RT-PCR and by *in situ *hybridization in chicken gonads from embryonic day-5 to adulthood. It depends both on the sexual determinism and on the L/R asymmetry pattern of gonad differentiation. This gene is specifically transcribed in somatic cells of the ovarian cortex involved in the formation of the follicles and in granulosa cells of the adult hen ovary. Its expression enlightens the profound remodeling of the ovarian cortex that occurs during follicle morphogenesis, an important phase of the ovarian differentiation which has not yet received much attention.

## Results

### Identification of differentially expressed genes in chicken embryonic ovaries using SSH

In order to identify unknown factors involved in early steps of the ovarian cortex differentiation, we performed a suppression subtractive hybridization screening (SSH) [37] to selected transcripts expressed in the chicken differentiating left ovary and underrepresented in the right gonad in which the cortex does not differentiate. The 8-day embryonic stage (E8) was chosen because the process of cortex development is at its beginning; meiosis has not yet started; and the gonads are easily dissected. We generated a left ovary cDNA-enriched library (LO-RO), by subtracting *Rsa*I-digested cDNAs from the left ovary (LO) with those of the right ovary (RO), and a right ovary cDNA-enriched library (RO-LO) by the opposite screening. Two hundred and fifty clones from the (LO-RO) library were pick-up randomly and submitted to differential hybridization screening. Macroarrays established with the PCR-amplified inserts were hybridized with labeled cDNA probes prepared with either the (LO-RO) or the (RO-LO) subtracted libraries. Asymmetry of expression was quantified by the ratio of (LO-RO)/(RO-LO) counts. We report here the study of one clone, OG43, which had a differential hybridization-screening ratio of 154, and a high expression in gonads, preferentially the ovaries, with left-right asymmetry. This clone was shown to correspond to an endogenous retroviral element not yet identified that we named *Ovex1 *(Ovary expressed, #1) in relation to its expression pattern.

### Sequence analysis of the *Ovex1* locus

**Figure 1:**
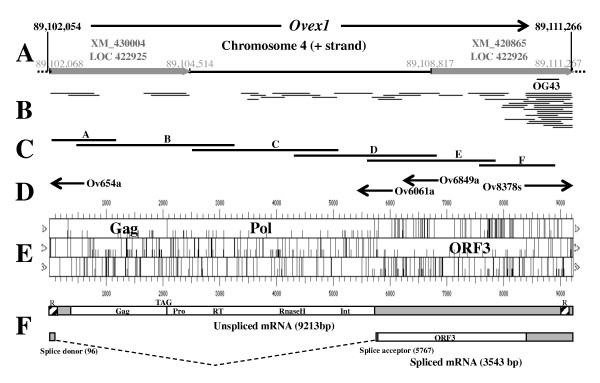
**Structure of the chicken *Ovex1 *endogenous retrovirus**. (**A**) Genomic position of *Ovex1*. LOC422925 and LOC422926 (May 2006 *Gallus gallus *v 2.1, galGal3 genome draft assembly) are indicated. The SSH-cloned OG43 cDNA fragment is shown. (**B**) Published ESTs (8-26-2007). (**C**) Overlapping RT-PCR fragments amplified from ovarian RNA used for cDNA sequencing. (**D**) Determination of mRNA ends by 5' and 3'RACE PCR (primers used and direction of the synthesis). (**E**) ORF map. (**F**) Transcription profile. Two types of mRNA are produced: a mRNA corresponding to the complete genomic sequence and a mRNA with one splicing event. Position of the splice donor and acceptor sites relative to the transcription start site is indicated between brackets, and the spliced out domain represented by a dotted line. Untranslated regions are shown in gray and the 5' and 3' imperfect repeats marked by (R). Encoded protein domains are indicated. Primer sequences are given in additional file 9 (Table S1, Primers and PCR conditions).

Identification of the insert of clone OG43 was performed by BLAT screening of the May 2006, v2.1 draft assembly of the chicken genome (galGal3). It revealed a 99.7% identity with the 3'-untranslated region (UTR) of a hypothetical gene [GenBank:XM_420865] (locus LOC422926), located on the chromosome 4 long arm (Fig. 1A). This gene, annotated as coding for a protein "similar to env", corresponds to the 3' region of the sequence given in Fig. 3, from nucleotide 6764.

**Figure 2:**
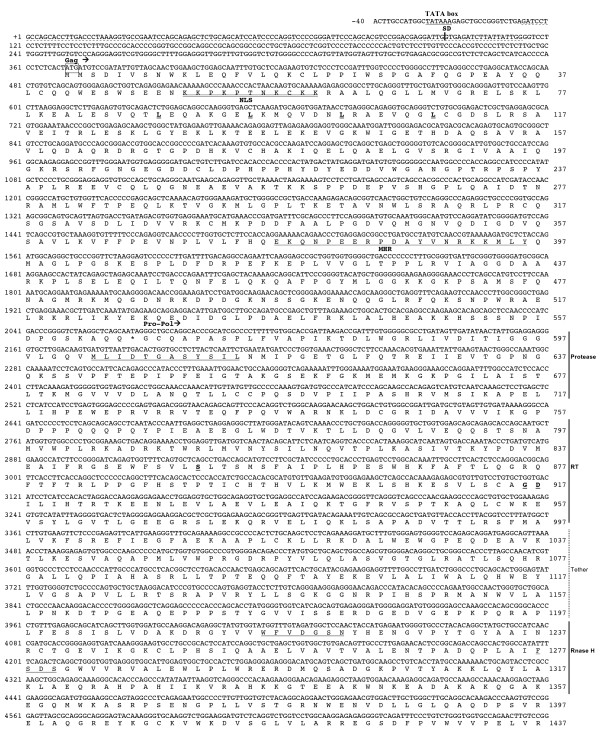
**Complete nucleotide sequence of chicken *Ovex1 *and predicted translation products (first part)**. See legend in Fig. 3.

Most of the expressed sequence tags (EST) corresponding to this locus have an ovarian origin. Some of them extend 5' from the locus (Fig. 1B), suggesting that transcription might start further upstream. Interestingly, the next locus upstream from LOC422926 in the galGal3 draft assembly, LOC422925, displays also a strict ovarian expression. It corresponds to a predicted gene, [GenBank:XM_430004], coding for a hypothetical protein of unknown nature (Fig. 1A) and extends from nucleotide 15 to 2461 of the sequence given in Fig. 2. None of the already published ESTs overlaps with the two loci. To examine if there might be a relationship between these two neighboring loci that have the same orientation and display the same specificity of expression, we tried to amplify overlapping cDNA fragments from one locus to the other by RT-PCR using embryonic ovary mRNA. The series of fragments obtained demonstrates that the two loci constitute in fact a single transcription unit (Fig. 1C).

The initiation cap site of this mRNA was determined by rapid amplification of the 5'cDNA-end method (5'RACE) using, in a first experiment, Ov6849a as antisense primer (Fig. 1D). Two sequences were amplified, indicating the existence of two types of mRNA: a genomic mRNA similar to DNA and a spliced subgenomic transcript lacking the 97–5766 sequence (Fig. 1F). Additional 5'RACE experiments confirmed this result, one with a primer (Ov6061a) located downstream from the acceptor splice site which allows only amplification of the short spliced transcript, and the second with a primer (Ov654a) located in the intron to amplify the unspliced mRNA. Both experiments gave the same 5'-terminal sequences, indicating that the cap site of the two mRNAs is presumably G_+1 _or A_+4 _(Fig. 2), a few bases upstream from the putative start of LOC422925. The cap site is located 23 nucleotides after a consensus TATA box (Fig. 2).

**Figure 3:**
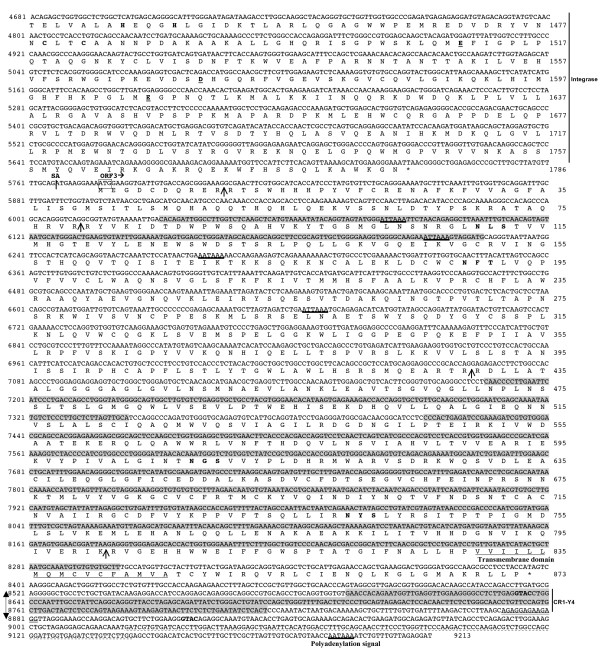
**Complete nucleotide sequence of chicken *Ovex1 *and predicted translation products (second part)**. Nucleotides are numbered on the left, from the most 5' cap site of the mRNA as position +1. The genomic sequence preceding the start of transcription is taken from the chicken genome draft assembly (galGal3). The 5' and 3' imperfect direct repeats are indicated by dashed underlines. Sequences identified as GGLTR11 and CR1-Y4 by RepeatMasker are on a gray background. The TATA box and polyadenylation signals (AATAAA and ATTAAA) are underlined. SD and SA indicate the splice donor and acceptor sites. The conceptual translation of *Gag*, *Pol *and *ORF3 *is given under the nucleotide sequence, and amino-acid positions indicated on the right. Putative translation start codons are boxed. Conserved motifs are underlined: the Gag nuclear localization signals (NLS), leucine zipper motif and major homology region (MHR). In Pro, the active site is underlined. In RT, position of the usually conserved aspartate residues is indicated in bold underlined characters. In the integrase, amino acids involved in the zinc-binding finger are in bold characters and residues of the degenerated DD35E motif bold and underlined. In the ORF3 protein, potential N-glycosylation sites are in bold letters, potential cleavage sites indicated by arrows, and the transmembrane domain underlined. In the 3' UTR, the 13-base polypurine tract is doubly underlined. The position of the SSH cDNA fragment is indicated by a double arrow and its *Rsa*I terminal sites (GTAC) printed in bold characters.

The mRNA polyadenylation site was identified by 3' RACE (Fig. 1D), using a forward primer common to both mRNAs (Ov8378s). The longest sequence obtained was polyadenylated at position 9213 (Fig. 3). Shorter sequences, polyadenylated at positions 9203 to 9211, were also found. The polyadenylation site is preceded by a consensus polyadenylation signal, AAUAAA (nt 9190 to 9195). The maximum size of the unspliced mRNA is 9213 bp and that of the spliced transcript 3543 bp (not taking into account the polyA tail). No other splicing was detected by RT-PCR amplification using various pairs of primers.

Surprisingly, in addition to the final polyadenylation signal, *Ovex1 *contains one AAUAAA and three AUUAAA hexamers followed by U and GU-rich elements, clustered in the region of nucleotides 6082 to 6669, which might constitute polyadenylation signals leading to premature transcription arrest. However, the efficiency of these signals *in vivo *is low as demonstrated by the RT-PCR amplification of cDNA fragments D, E, and F (Fig. 1C) and the existence of ESTs overcoming the signals (Fig. 1B). To verify if polyadenylation at these internal signals would affect differently the spliced and unspliced mRNAs we performed RT-PCRs using sense primers located upstream from the polyadenylation signals, either into the intron to amplify the unspliced mRNA, or across the splice site to amplify the spliced product. Several antisense primers, upstream or downstream from the four polyadenylation signals, were tested in both cases. The amplification of cDNAs overrunning the signals indicates that at least a substantial fraction of both types of mRNAs is transcribed unto the final polyadenylation site (additional file 1: Figure S2, Effect of the internal polyadenylation signals).

The complete sequence of chicken *Ovex1*, obtained by sequencing the cDNA fragments and the cloned RACE products, is given in Figs. 2 and 3. It is 99.9% identical to a region of the chicken genome draft assembly version v2.1 (galGal3, chr. 4, + strand, 89,102,054 to 89,111,266). Differences between these two sequences determined on different strains of *Gallus gallus*, the wild type Red Jungle fowl for the DNA and the domestic White Leghorn strain in our study, are exclusively single nucleotide substitutions.

Blat search in the recently sequenced genome of a passerine bird *Taeniopygia guttata *(zebra finch) revealed the presence of a sequence with 83% identity to chicken *Ovex1 *(taeGut1, chr. 4, + strand, 65,939,815 to 65,948,592). The sequence is incomplete, due to the presence of a sequencing gap of some 600 bp in this first version of the genome. This *Ovex1 *homolog is located on zebra finch chromosome 4, syntenic to chicken chromosome 4 long arm, between the *CD8 *and *SMYD1 *loci as for chicken *Ovex1*. Such conservation indicates that this sequence is the ortholog of chicken *Ovex1*. The sequence of zebra finch *Ovex1 *is given in additional file 2 (Figure S3, Partial zebra finch *Ovex1 *sequence). Conservation of the TATA box, the polyadenylation signal, and of sequences surrounding the donor and acceptor splice sites suggests that zebra finch *Ovex1 *is transcribed and spliced as chicken *Ovex1*.

The first two thirds of the *Ovex1 *sequence appear to be present as a single copy in chicken (galGal3) and zebra finch (taeGut1) genomes. In contrast, in both species the last third of *Ovex1 *contains sequences identified by RepeatMasker as the internal part of a multi-copy LTR-containing element, GGLTR11-int [36]. In addition, an imperfect antisense 3'-fragment of CR1-Y4, member of the large family of chicken CR1 non-LTR retrotransposons (LINE) [31,38], is included in the 3'-UTRs. These sequences are indicated in Fig. 3 and in additional file 2 (Figure S3, Partial zebra finch *Ovex1 *sequence).

### Repeated sequences

Endogenous retroviruses are usually bordered by two LTRs classically divided in U3-R-U5 regions. The 5' U3 region contains promoter and enhancer elements. Transcription is initiated at the 5' U3-R junction and terminates at the 3' R-U5 junction. We looked for repeated sequences in the chicken *Ovex1 *gene and flanking sequences. Direct repeats of 120–122-bp were found on each side of the gene, from nucleotide -6 to +114, 17-bp after the TATA box, on the 5'-side, and from nucleotide 9023 to 9144, 46-bp before the polyadenylation signal, on the 3'-side (Figs. 2 and 3). These repeats, which have only 73% identity, might be degenerated forms of the R region of previous LTRs. Identity of the zebra finch repeats is even lower (69%). No other repeated sequences corresponding to the U3 and U5 regions were found in the genomic sequence in and around the gene. No sequence complementary to the 3'-end of a chicken tRNA [39] that might be the primer tRNA binding site (PBS) required for reverse transcription was identified, thus precluding classification of *Ovex1 *according to this usual criterion. The 3'-UTR contains a 13-purine sequence that may correspond to the polypurine tract that precedes the 3'-U3 region in retroviruses (Fig. 3). Conservation of the 5'-UTRs between chicken and zebra finch is globally low (65%), but the sequence surrounding the splice donor site is well conserved. By contrast, the 3'-UTRs are very similar (80% identity score).

Chicken and zebra finch *Ovex1 *5'-proximal DNA sequences were screened to detect transcription factor binding sites, using the MatInspector program. Among putative responsive elements conserved with a similar position in the 5'-flanking regions, we identified a TATA box and sites for Ras-responsive element binding proteins, forkhead domain factors, estrogen-related receptors, GATA binding factors, and bicoid-like homeodomain transcription factors, in particular Pitx2 [40]. These sites are shown in additional file 3 (Figure S4, Alignment of chicken and zebra finch 5'-proximal DNA sequences).

### Open reading frames

As seen in Fig. 1E, the unspliced chicken mRNA contains three large uninterrupted open reading frames (ORF). The first two ORFs are contiguous in frame and separated by a stop-codon. The third one is in a different frame and non-overlapping. The spliced mRNA contains only the third ORF (Fig. 1F). The conceptual translation is given in Figs. 2 and 3 for chicken and in additional file 2 for zebra finch (Figure S3, Partial zebra finch *Ovex1 *sequence). Protein similarity queries using Blastp have shown that the unspliced mRNA potentially encodes Gag and Pro-Pol polyproteins and that the spliced mRNA encodes a protein that might be an envelope protein. Chicken and zebra finch *Ovex1 *display the same organization, which is typical of numerous retroviruses. Alignment of these proteins is shown in additional file 4 (Figure S5, Alignment of Ovex1 putative proteins).

#### Gag polyprotein

The *Gag *initiation codon is usually the first AUG after the cap site. In the chicken unspliced mRNA, the first AUG, in position 313, initiates potentially a 5-amino-acid hydrophobic peptide. Initiation may also occur at the next AUG downstream, by reinitiation or leaky scanning [41]. The next AUG is at position 370, in a correct context for initiation of the translation (CUCACUAUGAUGU). If translation starts effectively at this codon, the ORF would encode a 565-residue protein till the first stop-codon (nucleotide position 2065 to 2067) (Fig. 2). However, a second AUG, immediately following this initiation codon in a seemingly less favorable context, has been chosen as initiator for the hypothetical protein encoded by LOC422925. The putative protein was analyzed using Blastp. Gag polyproteins are constituted by the matrix, the capsid and the nucleocapsid domains. The C-terminal region (from the amino-acid residue 248) has 22% identity with the capsid domain of an epsilon-like retrovirus, Snakehead fish retrovirus (SnRV) [42]. The Ovex1 protein contains a sequence EKQNPEERPDAYVNRKKMLY corresponding to the major homology region (MHR) with the three underlined fundamental residues [43]. This region, which provides the interface for capsid binding and dimerization, is present in all retroviruses with the exception of the spumaviruses. No zinc finger domain was identified here, unlike in SnRV and most retroviruses except the spumaviruses. In the N-terminal region, no sequence similarity was detected with other Gag proteins, even with that of SnRV, but they do have some homology. As in SnRV, the protein N-terminus is presumably not myristylated, and the N-terminal region contains a polybasic sequence (residues 50 to 61) with two successive consensus nuclear localization signals, KKPK**PTNKCKK**R [44]. As in SnRV, the protein has a region of strong probability of coiled-coil structure [45], between residues 62 and 114, like in the rod domain of myosin-type proteins. In addition, the Ovex1 protein contains a L(X)_6_L(X)_6_L(X)_6_L leucine zipper motif (residues 88–109) seen in numerous regulatory proteins. Thus, this protein may be classified as a Gag polyprotein by analogy with that of the SnRV and from the position of its coding sequence preceding a *Pol *sequence, but its functionality remains to be demonstrated. The uninterrupted zebra finch Gag polyprotein is well conserved, with 90% sequence identity to chicken Gag, and contains the same functional domains (see additional file 4 (Figure S5, Alignment of Ovex1 putative proteins)).

#### Pro-Pol polyprotein

The second long ORF immediately follows the *Gag *amber codon in the same translational frame and is presumably translated by suppression of this codon as seen for SnRV, gamma- and epsilonretroviruses [46]. Translation results in a 1786-amino-acid Gag-Pro-Pol polyprotein unto the next stop-codon. Analysis of the conceptual translation of this ORF allows the identification of retroviral aspartyl protease (Pro) and Pol polyprotein domains: reverse transcriptase (RT), Rnase H, and integrase (IN) (Figs. 2 and 3). In zebra finch, the *Pol *region is only partially sequenced and most of the *Rnase H *domain is unknown. Chicken and zebra finch Pol sequences display 81% identity in their common regions and the same characteristics (see additional file 4 (Figure S5, Alignment of Ovex1 putative proteins)).

The aspartyl protease domain, approximately between residues 583 and 686, contains the active site MLIDTGASYSIL and presents 29 to 26% identity with the proteases of *Sphenodon *endogenous virus SpeV [47], immunodeficiency viruses and porcine endogenous retrovirus (PERV). As for SpeV, we note the absence of the conserved GRD/N motif present in all retroviral proteases, except for spumaviruses [48].

The RT domain, from residue 767 to 965, displays a similarity with RTs of foamy viruses, fish viruses: Walleye epidermal hyperplasia viruses WEHV 1 and 2 [49], Walleye dermal sarcoma virus (WDSV), SnRV (33–30% identity), and with the partially sequenced reptilian SpeV. However in chicken as in zebra finch Ovex1, several fundamental RT residues are not conserved, in particular two of the three aspartates that make up the catalytic active site [50], a difference resulting presumably in the loss of the enzyme activity.

The chicken Rnase H core domain, between amino-acid residues 1211 and 1357, is similar to those of SnRV (35% identity) and Moloney murine leukemia virus, MMLV (32%) with, in particular, the conserved WFVDGSN and FSDS motifs [51]. The distance between this sequence and the RT domain is consistent with the presence of the tether region that separates RT and Rnase H domains in vertebrate retroviral *Pol *genes [52].

The integrase (INT) has a similarity with those of MMLV, WEHV2, foamy viruses and SnRV. It contains a putative zinc finger: H(X)_3_H(X)_29_C(X)_2_C, as most retroviral integrases. The core domain, rve, between residues 1500 and 1647, is only partially conserved. Where other integrases have the catalytic triad D(X)_55–60_D(X)_35_E, the motif is E(X)_59_D(X)_35_E in chicken and E(X)_59_E(X)_35_E in zebra finch Ovex1. The difference is important since substitution of the first aspartate by a glutamate drastically impairs the integrase activity of Rous sarcoma and HIV viruses [53]. The C-terminus does not contain the consensus GPY/F motif but a WM**GPV**RV sequence that might be the degenerated remnant of this motif [35].

#### ORF3: an envelope protein?

The *Ovex1 *third ORF (*ORF3*) is located downstream from *Gag-Pol*. It is entirely contained in the second exon of the singly spliced transcript (Fig. 1F). A similar organization occurs for the envelope protein of gammaretroviruses. The ORF starts 11 bp after the splice site and encodes a putative protein of 873 amino acids. This ORF is potentially interrupted by the presence of the internal polyadenylation signals that do not appear very efficient *in vivo*, as demonstrated above. In zebra finch, the third ORF encodes an 874-amino-acid putative protein with 81% identity with the chicken protein and similar characteristics (see additional file 4 (Figure S5, Alignment of Ovex1 putative proteins)).

This region is quite complex. Segments of sequence on gray background in Fig. 3 were identified by RepeatMasker as weakly similar to GGLTR11-int, a consensus sequence quoted in Repbase as internal part of an ERV1 chicken endogenous retrovirus [36]. Nucleotide conservation with GGLTR11 is rather low (60%) and better-conserved copies of ERV1 can be found in the chicken genome. Furthermore, the first two GGLTR11-like sequences are separated by 30 nucleotides in GGLTR11 while located 960 nucleotides apart in *Ovex1*. The intervening region, not identified by RepeatMasker, is locally similar to sequences found in chromosome Z. A similar presence of GGLTR11-related sequences is detected in zebra finch *ORF3*, as seen in additional file 2 (Figure S3, Partial zebra finch *Ovex1 *sequence). The conceptual translation of GGLTR11 is given in additional file 5 (Figure S6, Structure and conservation of *ORF3*-encoded proteins). Unlike *Ovex1 ORF3*, it contains stop-codons and frameshift mutations.

Significant similarity of the ORF3 putative protein with other proteins was found only in an avian retrovirus, the Tetraonine endogenous retrovirus (TERV). This defective ERV of the ruffled grouse, *Bonasa umbellus*, a member of the galliform order as chicken, contains 5' and 3' LTRs, a Gag-like protein and a truncated region considered as an envelope TM domain [54]. The similarity between Ovex1 and TERV is limited to this envelope domain, with 52% identity over a 92-amino-acid region (residues 754 to 845) that can be extended upstream to amino acid 645, with stop-codons and frameshifts in the TERV sequence. This TERV region is closer to the consensus GGLTR11 than is the Ovex1 sequence. The main arguments sustaining the identity of the TERV envelope are its position just before the 3' LTR and some similarity with envelope proteins of MMLV and PERV. Identity of ORF3 with these retroviral envelopes is even more limited.

The protein potentially encoded by *ORF3 *is larger than prototypical retroviral envelope proteins. It contains two hydrophobic regions shown in additional file 5 (Figure S6, Structure and conservation of *ORF3*-encoded proteins). One is close to the N-terminus, and the second, located in the C-terminal part, corresponds to the identified transmembrane anchor and is preceded by a sequence susceptible to adopting a coiled-coil structure (residues 753 to 780). The cytoplasmic domain, C-terminal to the TM region is short. Retroviral envelopes are glycosylated. Four conserved potential N-glycosylation sites are present in the sequence. However, the protein does not seem to possess a signal peptide. Retroviral envelope precursors are cleaved between the surface and transmembrane domains at specific RX(R/K)R sites. The proteins encoded by *ORF3 *in chick and zebra finch contain conserved potential cleavage sites. However, neither these proteins nor TERV's contain the conserved CWLC sequence [55], the consensus immununosuppressive motif CKS17, and the disulfide bonded loop C(X)_6_CC that precede the TM region in many retroviruses [43,56]. Thus, despite its position and its homology with the TERV predicted envelope, the identity of the *ORF3 *product as an envelope protein is still uncertain, given its limited similarity with other retroviral envelope proteins.

### Single nucleotide polymorphisms (SNPs)

Differences between the sequence of chicken *Ovex1 *transcripts and the genomic galGal3 draft assembly are exclusively point substitutions reported in additional file 6 (Table S7, Polymorphisms in chicken *Ovex1 *sequences). Among 14 substitutions in *Gag*, *Pol *and *ORF3 *coding sequences, 12 are silent. These differences reflect the polymorphism between two types of *Gallus gallus *derived from a common ancestor: the wild Red Jungle fowl and the domestic White Leghorn strain. In addition, direct sequencing of RT-PCR products from pools of gonads allowed the detection of the presence of two nucleotides in variable proportions at some positions of the sequence. This heterogeneity is presumably due to polymorphism within the Leghorn chicken population. Some of these differences are also found in ESTs. In the sequence coding for Gag-Pol, these SNPs correspond to silent differences. In ORF3, conservative and non-conservative substitutions are observed.

### Presence of *Ovex1*-related sequences in other birds

**Figure 4:**
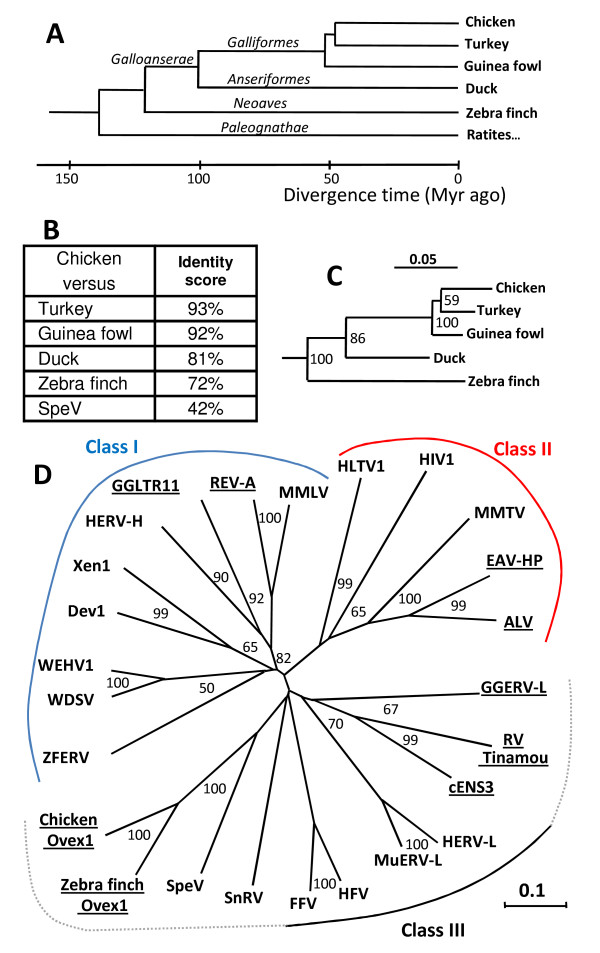
**RT-based phylogenetic analyses**. (**A**) Simplified phylogenetic relationship of modern bird lineage. Bayesian estimates of the divergence times (in million years, Myr) are based on mitochondrial gene data [68,91]. (**B**) Protein identity scores of avian Ovex1 RT fragments. The 133-amino-acid sequences of the RT fragments obtained by PCR from turkey, guinea fowl and duck were aligned with the homologous regions of chicken and zebra finch (Gag-Pol residues 768 to 900). The corresponding sequence of SpeV was added for comparison. The alignment, performed with ClustalW2, is shown in additional file 7 (Figure S8, Alignment of Ovex1 RT and homologous sequences). (**C**) Relationship of the five Ovex1 RT-fragment sequences. The phylogenetic tree and bootstrap values (1,000 iterations) were calculated by the neighbor-joining (NJ) method [85] using the QuickTree software and the tree drawn with NJplot. Branch lengths are proportional to substitution levels. The scale bar represents 5% divergence. Bootstrap percentages are indicated at the branch forks. (**D**) RT-based unrooted dendrogram of Ovex1 and representative retroviral elements. The alignment, corresponding to chicken Ovex1 Gag-Pol amino-acid residues 759 to 917, was performed with ClustalW2 with default settings and adjusted manually. It is given in additional file 7 (Figure S8, Alignment of Ovex1 RT and homologous sequences). The relationship and bootstrap values (1,000 iterations) were calculated by the neighbor-joining method with QuickTree and the unrooted dendrogram drawn with NJplot. Abbreviations and database accession numbers are given in Materials and methods. RTs of avian origin are underlined. Branch lengths are proportional to substitution levels. The scale bar represents 10% divergence. Bootstrap percentages are indicated at the branch forks when at least equal to 50%.

To investigate the presence of sequences related to *Ovex1 *in the genome of other birds, we performed PCR amplifications with primers corresponding to *Ovex1 Gag *and *RT *regions, using DNA from turkey (*Meleagris gallopavo*), guinea fowl (*Numida meleagris*) and duck (*Anas platyrhynchos*). Direct sequencing of the fragments gave unique sequences, corresponding to ORFs highly similar to those obtained from chicken. The 132-bp-long *Gag *fragment, which contains the nuclear localization signals, has no analog in nucleic-acid and protein databases. Conservation for turkey, guinea fowl and duck with respect to chicken is 98%, 96% and 92% at the nucleotide level and 100%, 100% and 98% at the protein level. The 400-bp-long RT fragments present 94%, 92% and 84% of nucleotide conservation. Although it is not proven that the amplified *Gag *and *Pol *sequences are linked in the DNA of turkey, guinea fowl and duck, the result suggests that *Ovex1 *orthologs exist in these birds. Alignment of the protein sequences is shown in additional file 4 (Figure S5, Alignment of *Ovex1 *protein sequences). Amino-acid identity scores for the RT fragments range from 93% between chicken and turkey to 76% between chicken and zebra finch (Fig. 4B). By comparison, identity with the closest known retrovirus, SpeV, is only 42%. The neighbor-joining tree based on the alignment of the five Ovex1 RT sequences (Fig. 4C) follows the bird phylogenetic relationship shown in Fig. 4A.

### Comparison with other retroviral elements

To classify *Ovex1 *amongst retroviral sequences according to the criteria defined by Jern [35], we may recall general characteristics. (i) The *Gag-Pro-Pol *coding sequences are in the same frame and translation of the Pro-Pol polyprotein results likely from the translational suppression of the *Gag *stop-codon, as in gamma-, epsilon- and intermediate epsilon-like retroviruses. (ii) The putative Gag protein contains no zinc finger, as in spumaviruses and spuma-related HERV-L and MuERV-L [43,57]. This is in contrast to the SnRV, which displays some similarity with Ovex1 Gag but has one zinc finger. (iii) No dUTPase domain was detected, unlike in MuERV-L. The absence of the integrase GPY/F motif is not discriminating as for spuma-like viruses, since a degenerated sequence may be present. A single splicing event and no obvious accessory ORFs were found in *Ovex1*, unlike in complex retroviruses like SnRV, WEHV and spumaviruses. In the analysis, it is necessary to distinguish the *Gag-Pol *and the *ORF3 *regions of *Ovex1*.

RT is the most conserved retroviral domain generally used for phylogenetic analysis, allowing detection of the relationship between distant elements [58]. We performed the alignment of a 159-amino-acid sequence of chicken and zebra finch putative RTs (Gag-Pol polyprotein residues 759 to 917) to that of representative retroviral elements and retroviruses, using ClustalW2 (see Additional file 7 (Figure S8, Alignment of Ovex1 RT and homologous sequences). The derived neighbor-joining unrooted dendrogram presented in Fig. 4D displays three groups of sequences. They correspond to the class I and class II of retroviral elements and to a third group more dispersed (similar to group I in [59]) that contains class III elements and the intermediate epsilon-like retrovirus SnRV [34,35]. Ovex1 RT is not closely related to any known avian retroviral sequence. On the basis of this analysis, Ovex1 does not belong to class II. Despite some similarity with the epsilonretroviruses WEHV and WDSV, it is not included either in class I elements. It appears more related to the third group of sequences. However, its relation with members of this group is not well defined. Ovex1 does not cluster with spumaviruses or spuma-related elements MuERV-L and HERV-L. It is not either similar to the Repbase GGERV-L element [36] or to avian sequences like ENS-3 (Chicken Embryonic Normal Stem cell gene 3) [60] and the bird Tinamou retrovirus [59]. Ovex1's closest relative is the *Sphenodon *endogenous virus, SpeV, found in an archaic reptile [47]. So far, only the *Pro *and *RT *domains of this endogenous virus are known, and they are the most similar to Ovex1. Ovex1 and SpeV constitute a distinct branch of the RT-based phylogenetic tree, close to the branch point of SnRV and spumaviruses. However, Ovex1 and SpeV are distantly related since their RT identity score is only 42%, which is not higher than between some members of different classes.

The second region of chicken and zebra finch *Ovex1*, corresponding to the third ORF, is partially related to GGLTR11, a class I ERV, which is not the case for *Pol*. Similarity with *Bonasa *TERV is limited to the transmembrane domain of the putative TERV's envelope. TERV is a defective endogenous retrovirus devoid of *Pol *that has been classified as an alpharetrovirus on the basis of its LTRs and *Gag *region but, according to the authors, the truncated *Env *might have a different origin [54]. Similarly in *Ovex1*, *ORF3 *and *Gag*-*Pol *might originate from different retroviruses.

### Analysis of chicken *Ovex1 *expression by RT-PCR

**Figure 5:**
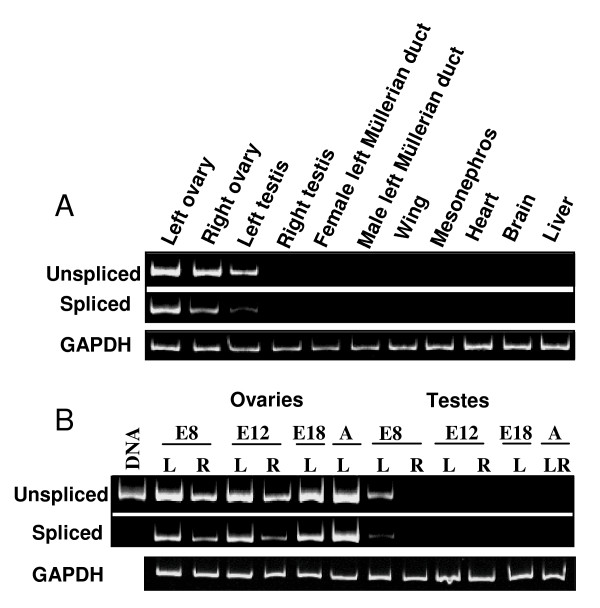
**Semi-quantitative RT-PCR analysis of *Ovex1 *expression in chicken tissues. (A**) Expression of *Ovex1 *in 8-day embryo tissues. RT-PCR was performed with pairs of primers designed to amplify specifically the unspliced mRNA (*GagPol*) or the spliced transcript (*ORF3*). Amplification of *GAPDH *transcripts was used as control for mRNA amounts. Primers and RT-PCR conditions are given in additional file 9 (Table S1, Primers and PCR conditions). A similar pattern of expression is observed in both cases. The level of transcripts is higher in the left ovary than in the right one and in the left testis. Expression is null in the right testis and in the other tissues tested, but for occasional traces in the wing. (**B**) Expression of *Ovex1 *in chicken gonads, as a function of age. The unspliced and spliced mRNAs were amplified from 8- and 12-day embryo left (L) or right (R) gonads, from 18-day embryo left gonads, and from adult (7 weeks) left ovaries or mixed (LR) testes. The identity of the RT-PCR amplified product was verified by sequencing. A control with chicken genomic DNA was performed in order to verify the absence of the deleted form in the genome and the specificity of amplification. The level of both types of mRNAs is up-regulated in the left ovary, weak in the right one, down-regulated after 8 days in the left testis, and null in the right one.

Semi-quantitative RT-PCR amplification of *Ovex1 *transcripts in various 8-day chicken embryo tissues shows that the unspliced *Gag-Pol *mRNA and the spliced *ORF3*-containing transcript are expressed in a similar manner (Fig. 5A). Expression is higher in the left ovary than in the right one, lower in the left testis, and absent in the right testis. Amplification is negative for the other tissues investigated (mesonephros, heart, brain, liver), but for traces in the wings. In female embryos, expression of both types of transcripts is asymmetrical at 8 and 12 days (Fig. 5B). The highest expression is found in the adult (left) ovary. The expression observed in the left testis at 8 days is down-regulated at 12 days and after, and the right testis remains negative.

### *In situ *hybridization

Expression of *Ovex1 *in chickens was examined by *in situ *hybridization using a probe corresponding to the *Pol *region and compared with that of other genes expressed in the gonads. In both sexes, the region of the presumptive gonad can be first identified at four days of incubation (E4) (Hamburger and Hamilton stage 24, HH24) by the expression of the Lim homeobox gene, *Lhx9*, in a restricted area of the mesonephros coelomic epithelium [4,61]. At this stage, neither the transcripts of *Ovex1 *nor those of the estrogen receptor alpha *ER-α *are detected in this region (not shown).

**Figure 6:**
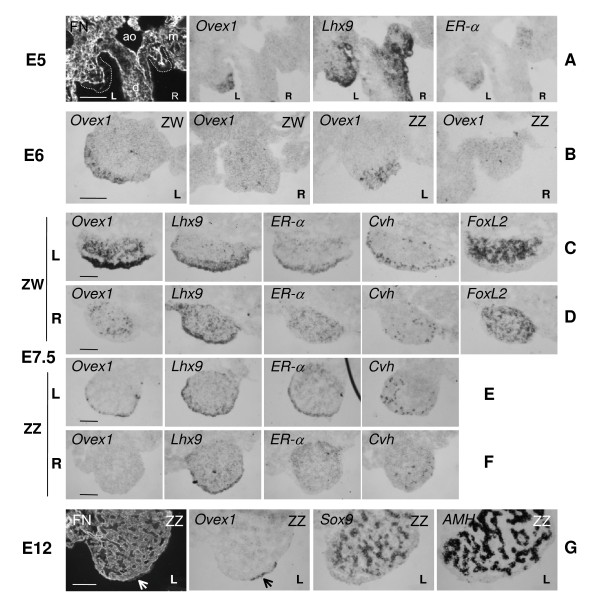
**Expression profiles of *Ovex1 *and gonad markers in embryonic gonads before and after sex determination**. Fibronectin (FN) is revealed by immunofluorescence and gene expression detected by *in situ *hybridization of digoxigenin-labeled RNA probes on transverse cryostat sections. (**A**) Immunostaining of fibronectin (FN) and expression of *Ovex1*, *Lhx9 *and *ER-α *in serial sections of a chick embryo (female) at embryonic day 5 (E5), (Hambuger and Hamilton stage HH27). Male embryos gave similar results. Gonads are outlined by a dashed line on the fibronectin image that corresponds to a double labeling of the section hybridized with *Ovex1*. m, mesonephros; d, dorsal mesentery; ao, aorta. (**B**) *Ovex1 *expression in left (L) and right (R), female (ZW) and male (ZZ) gonads at E6 (HH28). **C-F**: Expression of *Ovex1*, *Lhx9, ER-α, Cvh *and *FoxL2 *at E7.5 (HH32). (**C**) Left ovary. **(D**) Right female gonad. (**E**) Left testis. (**F**) Right testis. *FoxL2 *was not detected in testes. (**G**) Immunostaining of fibronectin (FN) and expression of *Ovex1*, *Sox9 *and *AMH *in E12 (HH38) left testis. Arrows indicate the epithelial region expressing *Ovex1*. Scale bars = 100 μm.

At E5 (HH27) (Fig. 6A), male and female gonads, morphologically indistinguishable, are protrusions at the surface of the mesonephroi. They comprise two territories: the outer epithelial area or "cortex", negative for fibronectin, and the inner region, or "medulla", containing irregular groups of cells separated by strands of fibronectin-positive material [4,7]. The two gonads are not identical: the left one is larger and has a thicker cortex. The pattern of expression of the studied markers is the same for male and female embryos. *Ovex1 *starts to be transcribed with a L/R asymmetry. Transcripts are present in the apical region of the cortex of left gonads, whereas they are not detected in right ones or in the mesonephros and surrounding tissues. *Lhx9 *is transcribed in the totality of the cortex of both gonads, and in part of the dorsal mesentery epithelium. At the same time, *ER-α *starts to be expressed asymmetrically in the cortex of left gonads and symmetrically in the medulla of the both, as reported [14,15].

At E6 (HH28) (Fig. 6B) in both sexes, the cortex is thicker in left gonads. In the female (ZW), *Ovex1 *is now detected in both gonads, but with a very dissimilar distribution. In the left ovary, it is expressed in the columnar cortical cells of the medioventral region, whereas in the right gonad a few *Ovex1*-expressing cells are scattered throughout the medulla. In the male (ZZ), expression of *Ovex1 *is visible in the medioventral region of the left testis cortex, whereas no expression is detected in the right gonad.

At E7.5 (HH31), the morphological L/R asymmetry of the female gonads is even more evident. In the left ovary (Fig. 6C), the thickening cortex, constituted of multiplying oogonia and somatic cells, is bordered by a single epithelial-cell layer. The medulla contains loosely connected cords of epithelial cells. *Ovex1 *is highly expressed in the cortical region including the surface epithelial cells, and also to a lower extent in the medullar cords. Likewise *Lhx9 *and *ER-α *are expressed in the cortex and more faintly in the medulla, but the patterns are not strictly identical. *Ovex1 *and *ER-α *transcripts are not detected at the lateral ends of the gonad, where the thickness of the cortex diminishes. Oogonia, identified by the chicken vasa homolog, *Cvh*, a germ cell-specific factor of the DEAD-box family [62], are mostly located in the depth of the cortex, although rarely some are dispersed in the medulla. If a germ-cell expression of *Ovex1 *cannot be fully excluded, it is clear at this stage that most of the cells that express *Ovex1 *in the left gonad are somatic cells and not germ cells. *FoxL2*, the female-specific forkhead transcription factor [28,63], is transcribed exclusively in the medulla in cordonal cells.

In the smaller right ovary (Fig. 6D), the cortex has not undergone the same development and the medulla represents the major part of the gonad. *Ovex1 *transcripts are completely absent from the cortical region but are present in some dispersed medullar cells. Similarly, *ER-α *is only expressed in the medulla as reported [9,10]. *Lhx9 *is expressed in the thin cortical region and in dispersed medullar cells, whereas *FoxL2 *is expressed in patches in the major part of the gonad but absent from the surface region. A few germ cells are present in the gonad.

At this stage, the morphological L/R asymmetry of the gonads is less obvious in males. Testicular differentiation becomes morphologically visible in both gonads. Sertoli cells, that start to express *Sox9 *and a high level of *AMH*, are clustering to form the sex cords [7]. A weak expression of *Ovex1 *is observed in the thin surface epithelium of the left testis (Fig. 6E), but none in the right one (Fig. 6F). No expression is detected in the medullas. *Lhx9*, by contrast, is expressed in the cortex of both gonads (Figs. 6E and 6F). Expression of *ER-α *is asymmetrical. Only the left testis displays a cortical expression whereas a faint symmetrical presence of transcripts is detected in both medullas (compare Figs. 6E and 6F), as reported [14,15]. Germ cells, more numerous in the left testis than in the right one, are dispersed in the gonads (Figs. 6E and 6F). At this stage, germ cells display a mostly peripheral distribution, a rather puzzling situation because Sertoli cells are clustering in the central part of the medulla to form the sex cords in which these germ cells are to be enclosed, as previously observed [4].

In the male, *Ovex1 *expression remains limited. At E12 (HH38), left and right testes appear morphologically rather similar. In both gonads, testis cords have formed into the medulla, containing germ cells and supporting Sertoli cells surrounded by a basal membrane. The male-specific marker *Sox9 *identifies the Sertoli cells that express also *AMH*. In the left testis, *Ovex1 *transcription is restricted to a small apical zone where the epithelium is thicker (Fig. 6G). It is still absent in the right testis (not shown). At E18, *Ovex1 *transcripts are no longer detected in the left testis (not shown). Disappearance of *Ovex1 *transcription in the left testis is coincident with that of *Pitx2c *[14] and *ER-α *[10].

**Figure 7:**
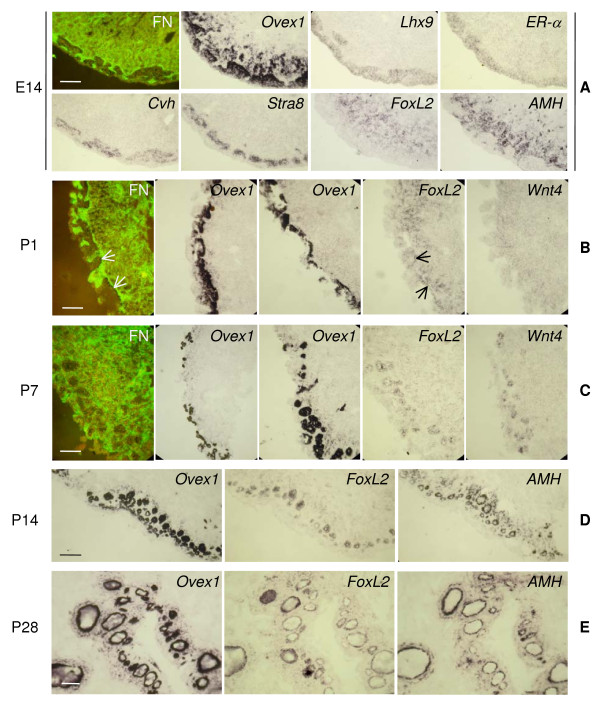
**Expression profiles of *Ovex1 *and gonad markers in the left ovary before and after folliculogenesis**. Fibronectin (FN) is revealed by immunofluorescence and gene expression detected by *in situ *hybridization of digoxigenin-labeled RNA probes on transverse cryostat sections. (**A**) Left ovary at E14. Immunostaining of fibronectin (FN) and expression of *Ovex1*, *Lhx9*, *ER-α, Cvh, Stra8, FoxL2 *and *AMH *in gonad serial sections. Fibronectin and *Cvh *are double labeling of the same section. (**B**) Left ovary at hatching (P1). Immunostaining of fibronectin (FN) and expression of *Ovex1 *(two aspects of the cortex desquamation process are presented), *FoxL2 *and *Wnt4*. Fibronectin and *FoxL2 *are double labeling of the same section. Arrows indicate *FoxL2*-positive cells located on the cortical side of the fibronectin cortico-medullar interface. (**C**) Left ovary 7 days after hatching (P7). Immunostaining of fibronectin (FN) and expression of *Ovex1 *(two aspects of the folliculogenesis are presented), *FoxL2 *and *Wnt4*. Fibronectin and *FoxL2 *are double labeling of the same section. (**D**) Left ovary at P14. Expression of *Ovex1, FoxL2 *and *AMH *in serial sections. (**E**) Left ovary at P28. Expression of *Ovex1, FoxL2 *and *AMH *in serial sections. Scale bars = 100 μm.

At E14 (Fig. 7A), the well-developed cortex of the left ovary, overlaid by a single epithelial-cell layer, is composed of fibronectin-negative regions separated by strands of fibronectin-positive material, often in continuity with the medulla, infiltrating sometimes up to the surface cell layer. The fibronectin-positive medulla is formed of groups of loosely connected cells and contains lacunae in its inner region. The interface between cortex and medulla is underlined by a thick deposit of fibronectin. The cortical fibronectin-negative nests contain clusters of germ cells and somatic cells. Germ cells are identified by the expression of *Cvh *and of the pre-meiotic factor *Stra8 *[64]. Somatic cells express *Lhx9*. *Ovex1 *is highly transcribed in cells located at the inner side of these cortical nests and faintly expressed in the gonad surface cell layer. At this stage, the high density of germ and somatic cells intermingled in the cortical nests does not allow to exclude that *Ovex1 *might be expressed in both cell types. Groups of *Ovex1*-positive cells are also dispersed within the subcortical region of the medulla. *Lhx9 *transcripts are totally absent from the medulla. The dispersed *FoxL2 *expression is limited to the medulla and *AMH *transcripts are located in the subcortical region of the medulla and in the fibronectin-positive cortical strands, as previously reported [4]. The degenerating right female gonad, relatively small and devoid of cortex, has no longer been studied.

Around hatching (P1) (Fig. 7B) occurs a dramatic remodeling of the left ovarian cortex. This is a prelude to folliculogenesis. The gonad surface cell layer, which has become negative for *Ovex1*, undergoes local disruptions leading to a sort of peeling. Exfoliation is initiated by apoptosis of superficial cells, as seen by TUNEL labeling (Additional file 8 (Figure S9, Detection of apoptotic cells in chicken ovary at hatching)). The desquamated surface of the gonad becomes jagged, with irregular cracks and protuberances. The phenomenon is emphasized by the intense expression of *Ovex1 *in cells that form a nearly continuous but irregular layer, especially visible in the bottom of the cracks. This layer appears to form a barrier resisting the desquamation process. At this stage, *FoxL2 *is expressed as previously in the medulla, more specifically in the juxtacortical region, but now also in some cells located on the cortical side of the fibronectin deposit that delimits cortex and medulla (shown by arrows). *Wnt4 *expression in the gonad is almost undetectable.

Folliculogenesis starts in the left ovary after hatching, and is completed by 22 days [65]. One week after hatching, at P7 (Fig. 7C), the limit between cortex and medulla becomes undefined. Small follicular structures expressing high levels of *Ovex1 *are observed near or at the surface of the desquamated ovary. It is also in these structures that a low *FoxL2 *expression is now visible and where *Wnt4 *starts to be expressed. These cells express also *AMH*, as illustrated in [4]. At previous stages, expression of this hormone was restricted to cells of the fibronectin-positive regions, as seen in Fig. 7A.

At P14 (Fig. 7D), follicles of various sizes are present at the periphery of the ovary, the smaller ones (the last formed) being the most external. They are constituted of a single oocyte surrounded by a layer of somatic cells. These follicular cells (also called granulosa cells) express *Ovex1 *at a high level, *FoxL2*, *AMH*, and in addition *Wnt4 *as shown in [4]. At this stage, folliculogenesis appears uncompleted because a layer of *Ovex1*-positive cells remains close to the surface of the gonad.

At P28 (Fig. 7E), the ovary that has acquired an important development is highly folded. Cells expressing *Ovex1*, *FoxL2*, and *AMH*, as well as *Wnt4 *(not shown) are essentially the granulosa cells of the follicles.

### Effect of the inhibition of estrogen synthesis on *Ovex1* expression

**Figure 8:**
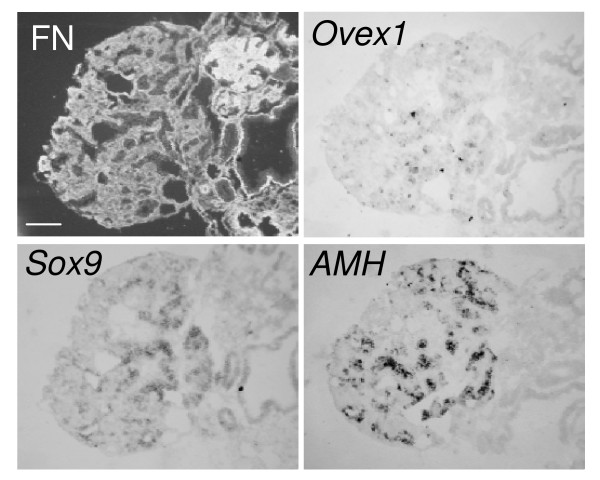
**Effect of fadrozole treatment on *Ovex1 *expression in the female left gonad at E14**. Chicken embryos were injected with fadrozole *in ovo *at E4. Fibronectin (FN) was revealed by immunofluorescence, and expression of *Ovex1, Sox9 *and *AMH *was detected by *in situ *hybridization of digoxigenin-labeled RNA probes on serial transverse cryostat sections of the masculinized left female gonad. Fibronectin and *Sox9 *are double labeling of the same section. Scale bar = 100 μm.

Estrogens play an essential role in ovarian differentiation. Aromatase, the key enzyme that converts androgens to estrogens, is expressed in female gonads from E5-E6. A possible involvement of estrogens in *Ovex1 *up-regulation after E6 in the left ovarian cortex and in the medulla of both female gonads, where the estrogen receptor ER-α is present, was thus investigated. Fadrozole (CGS 16949A), a non-steroidal aromatase inhibitor, was injected into the eggs at E4 to prevent estrogen synthesis, and the embryonic gonads examined at E14. Fadrozole treatment has been shown to lead to female to male sex reversal. The right gonad differentiates into a testis and the left one into an ovotestis or a testis. This is characterized by impairment of cortex development, decrease of aromatase synthesis, and formation of testicular cords expressing the testicular marker *SOX9 *and elevated levels of *AMH *and *DMRT1 *transcripts [22,27,66,67]. By comparison with the E14 control ovary (Fig. 7A), it is evident in Fig. 8 that the left gonad of the fadrozole-treated female is masculinized, with a thin cortical region and important medulla containing epithelial cords and lacunae. *Ovex1 *expression is extremely reduced in this gonad in the medulla as in the remnants of the cortical region. Conversely, *Sox9*, which is not normally transcribed in female gonads, and *AMH *are expressed in internal epithelial structures that resemble testis cords.

## Discussion

### Characterization of *Ovex1 *as endogenous retrovirus

The SSH technique used in this study was designed to identify genes transcribed preferentially in the chicken embryo left ovary and thus potentially involved in the differentiation of this gonad. It revealed the existence of a locus that we called *Ovex1 *according to its expression pattern. *Ovex1 *has a structure similar to that of simple endogenous retroviruses, with ORFs coding for potential proteins related to retroviral Gag and Pro-Pol polyproteins and a third one (ORF3) which displays only a faint similarity with retroviral envelope proteins. In the chicken ovary a full-length genomic mRNA and a singly spliced subgenomic transcript encoding only the third protein are present. Translation of the *Pro-Pol *ORF results presumably from read-through of the *Gag *stop-codon, like in gamma, epsilon and intermediate epsilon-like retroviruses.

A homologous sequence was found in the genome of zebra finch, a distantly related bird species. The location of these sequences, at similar positions in synthenic chromosomes, indicates an orthologous relationship. Partial sequences corresponding to *Gag *and *Pol *regions were also detected in turkey, guinea fowl and duck, suggesting the presence of similar genes in these species. Protein similarity of the five Ovex1 RT fragments follows the phylogenetic relationship of the bird species. This relation is indicative of the vertical transmission of an ERV integrated into the genome of a common ancestor of these birds, therefore before the split between Galloanserae (chicken, turkey, guinea fowl, duck...) and Neoaves (zebra finch...) some 122 Myr (million years) ago [68]. Investigation for similar sequences in Paleognathae (tinamou and ratites), which diverged from the lineage leading to other birds 139 Myr ago, would be necessary to ascertain the precise time of insertion.

Available data indicate that only one copy of the *Ovex1 Gag*-*Pol *region exists in the genome of chicken and zebra finch, in contrast to most ERVs that constitute families of related sequences derived from an initial integrated virus. This region is not closely related to other known avian retroviruses. According to the RT-based phylogenetic analysis, *Ovex1 *is mostly related to SnRV and class III retroviral elements but its relationship with these elements is very distant. Its closest relative is SpeV, an endogenous retrovirus of the tuatara (*Sphenodon)*, last representative of an archaic reptilian lineage that already existed 220 Myr ago [69]. However the identity is rather low. In SpeV, only a part of the *Pro *and *RT *domains have been sequenced and the comparison is limited. More information would be required to confirm the relationship of these ERVs.

In contrast, the region of *Ovex 1 ORF3 *and the 3'UTR contain sequences similar to an ERV I repeated element and a fragment of CR1 that are similarly located in chicken and zebra finch DNA. This means that *Ovex1 *is likely a composite element and that the recombination events occurred in an ancestral genome before divergence of the two bird clades 122 Myr ago. Such a chimerism resulting from exchange of *Pol *and *Env *is frequently observed in the ERVs [56].

The obvious deficiency of chicken and zebra finch *Ovex1 *in comparison with other ERVs is the absence of identifiable LTRs. However, two imperfect direct repeats, located in the regions at the start and end of the transcription unit are the presumable remnants of former LTRs. Retroviral LTRs, generated from a single template during reverse transcription of RNA into DNA, are identical at the time of integration into the host genome. Over time, they will diverge in sequence because of mutations occurring during cellular DNA replication. Differences between the two repeats can be used to estimate the time that has elapsed since the ERV integration [32]. The lack of full LTRs and the high divergence of the remaining 5' and 3' repeats are consistent with the ancient origin of *Ovex1*.

The great majority of ERVs, having suffered random mutations since their insertion, have accumulated deletions, frameshifts and stop-codons that interrupt their ORFs [70]. In chicken *Ovex1*, all three ORFs are uninterrupted (though small deletions or local frameshifts might have remained undetected) and in zebra finch at least two of them. This is a rare event, unless the ERV has a very recent origin, which is not the case. *In vivo *translation of *Ovex1 *transcripts remains to demonstrate. However, conservation of the ORFs suggests the existence of a selection pressure acting to retain not only the RNA but also the encoded proteins. The effect of selection can be revealed by comparing the rates of synonymous (dS) and nonsynonymous (dN) nucleotide substitutions in chicken and zebra finch coding sequences [71]. dN/dS ratios <1 indicate the effect of a purifying selection acting to eliminate divergent protein sequences. *Gag *is under a strong purifying selection, with a dN/dS ratio of 0.08, close to the mean value (0.085) for genes located on macrochromosomes [72]. *Pol *and *ORF3*, with dN/dS ratios of 0.18 and 0.16, display less constraint in the purifying selection. In addition, we detected a certain number of single nucleotide substitutions between the wild Red Jungle fowl and the White Leghorn chicken, a domestic strain derived from the same *Gallus gallus *ancestor after some 10,000 years of domestication [31]. Most of these substitutions are silent (as seen in additional file 6 (Table S7, Polymorphisms in chicken *Ovex1 *sequences)). This is in support of an ongoing purifying selection that appears more stringent for *Gag *and *Pol *than for *ORF3*.

*Ovex1*, devoid of homologous LTRs, is likely unable to propagate its sequence. Gag and Pol have retained enough conservation to be identified, but are nevertheless considerably different from proteins of other retroviruses. Important amino-acid residues implicated in viral enzyme activity are not present in chicken and zebra finch Ovex1 sequences whereas they are conserved in all retroviruses including Ovex1's closest relative, SpeV. RT and Int, if translated, are presumably inactive. The protein encoded by *ORF3*, if it is an envelope protein, appears also highly defective. Conservation of uninterrupted retroviral ORFs during more than 100 Myr despite the presence of a high number of presumably invalidating mutations can hardly occur just by chance and might indicate that these proteins fulfill some unknown biological function and are useful to the host.

### Expression of *Ovex1 *in chicken gonads

The *in situ *hybridization study shows that expression of *Ovex1 *is restricted to specific cells of the gonads and thus tightly controlled. This expression is characterized by L/R asymmetry and is dependent on sex differentiation. In both sexes, *Ovex1 *transcription is initiated at E5 in the cortex of left gonads before the onset of evident sex differentiation. At this stage, asymmetry of the gonads is already determined by the asymmetrical expression of *Pitx2c *in the epithelium of the left coelomic cavity, including the genital ridge [14]. In both sexes, Pitx2c inhibition of retinoic acid synthesis allows expression of *ER-α *and *SF-1 *and cortical cell proliferation in the left gonad [15]. The patterns of expression of *Pitx2c *[15] and *Ovex1 *at E5 and their transient expression in the left testis are rather similar, and it is plausible that Pitx2c controls *Ovex1 *expression, directly or indirectly. In this context, the presence of a conserved Pitx2 responsive element in the promoter region of chicken and zebra finch *Ovex1 *is interesting and deserves further study.

From E6 thereon, aromatase, the key enzyme of estrogen synthesis, is present in female gonads, not in male ones. Consequently, in female embryos, the cortex of the left gonad and both medullas, where the estrogen receptor ER-α is present, become direct physiological targets of estrogens. After E6 in the female, an increased and sustained transcription of *Ovex1 *occurs in the cortex of the left ovary and a low expression is observed in the medulla of both gonads. In the male, the cortical region of the left testis able to express *Ovex1 *becomes more and more limited. As *Pitx2c *[14], *Ovex1 *is no longer expressed at E18. The absence of *Ovex1 *transcription in testicular medullas suggests that estrogen stimulation might be required for the medullar expression. Estrogen deprivation by fadrozole treatment leads to the masculinization of female gonads with an inhibition of the left ovarian cortex development and the apparition in the medulla of epithelial structures analogous to testis cords. *Ovex1 *expression is strongly decreased in the medulla as well as in remnants of the cortical region, showing the requirement for estrogens to get a sustained expression. This effect might be due to a direct action of estrogens on the *Ovex1 *promoter but reflects also the role of the hormone in proliferation and/or differentiation of cortical and medullar cells able to express *Ovex1*.

During the embryonic differentiation of the ovary, most of the *Ovex1*-expressing cells are tightly associated with germ cells in the left cortex (or in close vicinity in the subcortical medulla). After hatching, they constitute the granulosa cell layer surrounding the oocyte in the forming follicles. This is in contrast with other markers of the granulosa like *FoxL2*, *AMH *and *Wnt4 *[4], which are not expressed in cortical cells during embryogenesis, but in the medullar compartment, and start to be expressed in granulosa cells only when follicles form. The continuity of expression of *Ovex1 *makes this factor an interesting marker of the filiation between cortical somatic cells and granulosa cells.

The expression of *Ovex1 *enlightens the dramatic remodeling of the ovarian cortical surface that occurs around hatching preceding the folliculogenesis. The previously compact cortex, which contained nests of tightly packed meiotic germ cells and somatic cells covered by a continuous surface cell layer, suffers an important morphological change. The surface cell layer loses *Ovex1 *expression; patches of cells undergo apoptosis, and local disruptions lead to an exfoliation of the superficial region of the cortex. *Ovex1*-expressing cells appear to constitute a barrier resisting the desquamation process before follicle formation. A more limited phenomenon has been observed by scanning electron microscopy in human and mouse ovaries. The extrusion of germ cells and satellite somatic cells through temporary breaks of the ovarian surface epithelium under the pressure of the subjacent highly proliferating tissue has been reported [73].

### Putative roles of *Ovex1 *in the gonads

*Ovex1 *expression constitutes an interesting marker of ovarian morphogenesis in chicken, but can it be an actor in this process? In the male, the transient *Ovex1 *transcription in the left testis is dispensable for testis function since the right gonad becomes a functional testis despite the absence of *Ovex1 *expression. The same conclusion cannot be drawn for female gonads.

ERV transcription in vertebrates is not an exception and displays variable tissue specificity [33,74]. In mammals, expression of several ERV envelopes is detected in various cancer cells, in particular ovarian cancers [75] and in several normal tissues [55,76]. However, the role of these retroviral proteins in vertebrate cells – if they are translated – is not often known. The unique but prototypical example is the recruitment of the fusogenic properties of retroviral envelopes for the specific mammalian function of placenta morphogenesis. HERV-W and HERV-FRD envelope proteins, named syncytin 1 and 2, are responsible for trophoblast cell fusion in placenta syncytiotrophoblast formation in humans [77,78]. Envelope proteins of various retroviruses have been independently recruited to fulfill the same function in other mammals [79,80]. However, it does not seem that embryonic ovary differentiation involves cell fusion. Another positive role of ERVs for the vertebrate host is protecting cells against infection by viruses of the same family. Endogenous envelope proteins saturate viral cell receptors, thus preventing exogenous virus entry. In addition, the Gag protein of MuERV has been shown to be closely related to the murine Fv1 gene that controls the replication of MMLV and prevents disease in mice infected by this retrovirus [43]. Such a role might be useful in the ovary.

It is noticeable that *Ovex1 *is not the only retroviral sequence expressed in the embryonic chicken ovary. *FET-1 *(female expressed transcript 1), a W-chromosome gene asymmetrically expressed in the left ovarian cortex between E4.5 and E6 has also a retroviral origin [2,11]. We found that it is similar to the consensus GGLTR7A retrovirus (described in RepBase) [36] over 87% of its length and encodes potentially an Env-like protein with similarity to the HERV-FRD Env protein (syncytin 2). A common role and/or a common regulation of *FET-1 *and *Ovex1 *might be investigated. To speculate further about the function of *Ovex1 *(and *FET-1*) in the ovary, it would be necessary first to establish whether the encoded proteins are actually translated, and if so, whether they retain viral functions, in particular if the putative envelope possess fusogenic properties. Alternatively, these proteins might have acquired new functions. The presence of nuclear localization signals and of a leucine zipper motif in Ovex1 Gag protein suggests a possible role in nuclear regulations.

In a recent screening for ESTs present in adult hen ovarian follicles, an EST [GenBank:EC912004] that corresponds to *Ovex1 *has been found to be expressed at a rate 6-time lower in hen ovaries of a chicken strain selected for its high egg production, compared with another strain with low egg production [81]. *Ovex1*, which is expressed in supporting somatic cells in close contact with germ cells in the embryonic cortex and in follicles up to adulthood, might play a role in regulating hen fertility at different stages: germ cell proliferation, meiosis, folliculogenesis, follicular survival or rate of follicle recruitment.

## Conclusion

*Ovex1 *is an ERV present in both chicken and zebra finch genomes. Similar sequences are detected in three other domestic birds. Sequencing of more bird and reptile genomes will presumably enlarge the family of *Ovex1*-containing species. Unlike most ERVs that are represented in the host genome by a family of related sequences, most of the *Ovex1 *sequence (*Gag *and *Pol *regions) exists as a unique copy in the genome of chicken and zebra finch. However, these genomes are not fully investigated and random divergence of elements inserted more than 100 Myr ago may impair their identification. The conserved copy of this ERV might have been preserved because of a specific function. Specifically expressed in the gonads, *Ovex1 *constitutes an interesting marker of the granulosa cell lineage useful for the study of ovarian morphogenesis. The future objective will be to determine if it is only a witness or if it is an actor. The involvement of a retroviral element in crucial processes of the ovarian development would be very exciting.

## Methods

### RNA preparation

Commercial White Leghorn chicken eggs were incubated at 38°C. Development stages (HH) are defined according to Hamburger and Hamilton [82]. Embryonic left and right gonads were dissected, collected individually into RNA-later solution (Qiagen), and stored at -80°C. Young embryos (up to E8) were genetically sexed by PCR [4] on DNA purified from extragonadal tissues with the NucleoSpin tissue kit (Macherey-Nagel). Total RNA was purified from pools of male or female, left or right gonads (35 gonads, about 18 mg of tissues for E8 embryos), using the RNeasy RNA mini extraction kit (Qiagen), with DNase treatment.

### Suppression Subtractive Hybridization (SSH)

The suppression subtractive hybridization (SSH) technique [37] was used to select transcripts expressed at a higher level in the left ovary than in the right one of E8 female chicken embryos. The cDNAs were prepared and amplified from total RNA of left or right ovaries, using the SMART PCR cDNA synthesis kit (BD Biosciences Clontech) according to the manufacturer's instructions. To generate the first strand, 1 μg of total RNA was reverse-transcribed with an oligo-dT containing primer (CDS), using PowerScript reverse transcriptase (BD Biosciences Clontech). Bicatenar cDNA was produced using the SMART II 5'-anchored primer and amplified by PCR for 15 cycles. SSH was performed with the PCR-Select cDNA Subtraction kit (BD Biosciences Clontech) according to the user's manual. The two amplified cDNA populations were digested with the restriction enzyme *Rsa*I. After digestion, the left ovary cDNAs (LO) were subtracted against the right ovary cDNAs (RO), giving a cDNA pool enriched in left ovary transcripts (LO-RO). Conversely, the right ovary cDNAs were subtracted against the left ovary cDNAs, giving a cDNA pool enriched in right ovary transcripts (RO-LO). The efficiency of normalization and subtraction was assayed by comparing the abundance of the constitutively expressed glyceraldehyde 3-phosphate dehydrogenase cDNA in initial and subtracted cDNA pools, using chicken-specific PCR primers (cGAPDHs: 5'ACCACTGTCCATGCCATCAC3', cGAPDHa: 5'TCCACAACACGGTTGCTGTA3').

### Cloning of the subtracted cDNA fragments

(LO-RO) subtracted cDNA fragments were cloned into pGEMT-easy plasmid (Promega), to construct a (LO-RO) subtracted cDNA library. Two hundred and fifty individual colonies were randomly selected, grown in 96-well plates in 100 μL LB medium plus ampicillin for 14 hours at 37°C and frozen at -80°C after addition of 10% glycerol.

### Differential hybridization screening

Differential hybridization screening was performed to identify the cDNA clones displaying the greatest asymmetry of expression between left and right gonads. A procedure adapted from the PCR-Select differential screening kit user's manual (BD Biosciences Clontech) was used with the following differences. The (LO-RO) cDNA clone library was used to prepare macroarrays. The insert of each clone was PCR-amplified, using PCR-Select nested primers 1 (5'TCGAGCGGCCGCCCGGGCAGGT3') and 2R (5'AGCGTGGTCGCGGCCGAGGT3'). Four identical macroarrays were prepared by spotting 2-μL aliquots of each PCR reaction mixture on nylon membranes (Hybond-N^+^, Amersham Pharmacia Biotech). Denaturation of the DNA was achieved by blotting the membranes onto 0.5 M NaOH, 1.5 M NaCl-impregnated Whatman 3 MM paper for 2 min, followed by neutralization with 0.5 M Tris-HCl pH7.4, 1.5 M NaCl for 5 min and 3xSSC for 5 min, under the same conditions. Dry membranes were then exposed to ultraviolet light (0.6 J/cm^2^) to perform DNA cross-linking. The membranes were hybridized with radioactive probes corresponding to the subtracted and non-subtracted cDNA pools: LO-RO, RO-LO, LO, and RO. ^32^P labeled probes were prepared by random priming (Invitrogen Random primers DNA labeling system). Hybridization was performed as described previously [83]. Denatured herring sperm DNA (100 μg/mL) and 3 μg/mL each of PCR-Select nested primers 1 and 2R, anti-nested primer 1: (5'ACCTGCCCGGGCGGCCGCTCGA3') and anti-nested primer 2R (5'ACCTCGGCCGCGACCACGCT3') were added to the hybridization solution to prevent unspecific hybridization of the PCR-Select adaptator sequences. Membranes were subjected to autoradiography and the intensity of the spots quantified using the ImageJ software. The clones displaying the highest ratios of (LO-RO) versus (RO-LO) hybridization were selected. The inserts were sequenced by automated fluorescence sequencing (MWG Biotech France), using pGEMT-specific primers.

### Determination of the complete *Ovex1 *sequence

Primers derived from the genomic sequence were used to amplify by RT-PCR overlapping cDNA fragments from E8 left ovary RNA. Reverse transcription was performed by random priming on 2 μg total RNA using SuperScript II Rnase H^- ^reverse transcriptase (Invitrogen). PCR amplification was performed on 1/20th of the cDNA product (equivalent to 100 ng of RNA), with 10 μg/mL of the PCR primer pairs listed in additional file 9 (Table S1, Primers and PCR conditions). The mixture contained 67 mM Tris-HCl (pH8.8), 17 mM (NH_4_)_2_SO_4_, 6.7 mM MgCl2, 10 mM 2-mercaptoethanol, 6.7 μM EDTA, 10% (v/v) dimethylsulphoxide, 0.5 mM each of the four dNTP, and 2 units of EurobioTaq polymerase (Eurobio) in a final volume of 50 μL. Hot start was performed by addition of the Taq polymerase after 5 min of preincubation at 80° and followed by 30 cycles of amplification (45 s at 95°, 45 s at 57°, and 180 s at 72°). After analysis for purity of the PCR product by polyacrylamide gel electrophoresis, the PCR reaction mixture was treated with ExoSap-It (USB) to remove primers and nucleotides and used for direct sequencing of the fragments in both directions (MWG Biotech France).

### Determination of cDNA ends

5' and 3' cDNA fragments were isolated by the rapid amplification of cDNA end methods (5'RACE and 3'RACE) using total RNA purified from E12 left ovaries. Amplification was performed with the SMART RACE cDNA amplification kit (BD Biosciences Clontech), according to the manufacturer's instructions, using SMART universal primers and the gene specific primers indicated in additional file 9 (Table S1, Primers and PCR conditions). The amplification products were analyzed by electrophoresis on agarose gel and the main band, purified with NucleoSpin extract II (Macherey Nagel), was cloned into pGEMT-easy vector (Promega). At least 5 independent recombinant clones were sequenced in each experiment.

### Amplification of *Ovex1*-related sequences from other domestic fowls

Turkey, guinea fowl and duck DNAs were prepared from muscular tissues. PCR amplification was carried out with primers and conditions indicated in additional file 9 (Table S1, Primers and PCR conditions). PCR fragments were sequenced directly.

### Computer sequence analysis

DNA sequence searches in the chicken (galGal3) and zebra finch (taeGut1) genome databases were performed with BLAT program . Nucleic acid and protein similarity was detected with the BLAST server at NCBI . Mobile elements were identified by RepeatMasker  based on the RepBase database [36]. Searches for the PBS were carried out using chicken tRNA sequence data [39]. Promoter screening for transcription factor responsive elements was carried out with the MatInspector program . Sequence alignments were performed with ClustalW2 multialignment program [84], with default settings  and adjusted manually. The phylogenetic trees and bootstrap values were calculated using the Neighbor-Joining method [85] with QuickTree software [86] and drawn with the NJplot software [87]. The ORF map and the hydrophobicity plot calculated according to Kyte and Doolittle [88] were created with the DNA Strider program [89]. Protein structure was analyzed with Psort version II  and N-glycosylation sites predicted by the NetNGlyc 1.0 Server . Synonymous and non-synonymous substitution rates corrected for multiple substitutions were determined using the SNAP program [90]. Quantification of photographic spot intensity was carried out with Image processing and analysis in Java (ImageJ 1.33) .

### Database accession numbers and abbreviations

Complete nucleotide sequences of the chicken *Ovex1 *unspliced and spliced mRNAs (splice variant 1) were deposited under accessions [GenBank:FJ406461] and [GenBank:FJ406462]. Partial *Gag *sequences of turkey, guinea fowl and duck are respectively [GenBank:FJ423166, GenBank:FJ423167, GenBank:FJ423168] and partial *RT *sequences [GenBank:FJ423169, GenBank:FJ423170, GenBank:FJ423171].

Abbreviations and database accession numbers of other sequences referred to in the text and figures are as follows: ALV, Avian leukosis virus, [GenBank:NC_001408]; cENS3, Pol-like protein ENS-3, [GenBank:NP_989963]; Dev1, Dendrobates ventrimaculatus ERV 1, [EMBL:X95795]; EAV-HP, Avian endogenous retrovirus EAV-HP, [EMBL:AJ292966]; FFV, Feline foamy virus, [GenBank:NP_056914]; GGERV-L_C_, ERV3 endogenous retrovirus from chicken, RepBase [36], RT (nt 2312–2785); GGLTR11-int, ERV1 endogenous retrovirus from chicken, RepBase [36], RT (nt 1686–2183), Env (nt 4126–5472); HERV-H, Human endogenous retrovirus HERV-H/env62, [EMBL:AJ289709]; HERV-L, Human endogenous retroviral element HERV-L, [EMBL:X89211]; HFV, Human foamy virus, [GenBank:NC_001736]; HIV1, Human immunodeficiency virus 1, [GenBank:NC_001802]; HTLV1, Human T-lymphotropic virus 1, [GenBank:NC_001436]; MMLV, Moloney murine leukemia virus (Pol polyprotein), [Swiss-Prot:P03355]; MMLV, Moloney murine leukemia virus (Env polyprotein), [Swiss-Prot:P03385]; MMTV, Mouse mammary tumor virus, [GenBank:NC_001503]; MuERV-L, Mus musculus endogenous retroviral sequence MuERV-L, [EMBL:Y12713]; PERV, Porcine endogenous retrovirus, [EMBL:AJ279057]; REV-A, Reticuloendotheliosis virus isolate REV-A, [GenBank:DQ237900]; RV_Tinamou, RV-Tinamou partial mRNA for polyprotein, [EMBL:AJ225235]; SnRV, Snakehead retrovirus, [GenBank:NC_001724]; SpeV, Sphenodon endogenous retroviral gene for protease and reverse transcriptase, [EMBL:X85037]; TERV, Tetraonine endogenous retrovirus, [GenBank:AF289082]; WDSV, Walleye dermal sarcoma virus, [GenBank:NC_001867]; WEHV1, Walleye epidermal hyperplasia virus type 1, [GenBank:AF133051]; WEHV2, Walleye epidermal hyperplasia virus type 2, [GenBank:AF133052]; Xen1, Xenopus laevis endogenous retrovirus Xen1, [EMBL:AJ506107]; ZFERV, Danio rerio endogenous retrovirus ZFERV, [GenBank:AF503912].

### Gene expression analysis by semi-quantitative RT-PCR

Semi-quantitative RT-PCR was carried out to determine the specificity of expression of *Ovex1*. Experimental conditions were as described above, except for the use of 1/100^th ^of the RT product (equivalent to 20 ng RNA). The number of PCR cycles was limited and optimized in each case. Primer pairs, hybridization temperatures and number of cycles are given in additional file 9 (Table S1, Primers and PCR conditions). Elongation at 72° was for 75 s. PCR products were analyzed by electrophoresis on 10% polyacrylamide gels. Controls without reverse transcriptase were negative.

### *In situ *hybridization

*In situ *hybridization was performed on Leghorn gonad cryostat sections as described previously [7], using antisense digoxigenin-labeled riboprobes. *Lhx9 *and *Cvh *probes have already been described [4]. Other probes were prepared from cloned RT-PCR fragments, according to the same procedure. The *FoxL2*, *Stra8 *and *ER-α *probes correspond respectively to [GenBank:AY155534] (nucleotides 1 to 330), [GenBank:XM_416179] (nt 121 to 825) and [EMBL:X03805] (nt 827 to1752). For *Ovex1*, a *Pol *probe corresponding to nucleotides 2511 to 3245 was used in the experiments reported. Hybridization of a 3'-UTR probe (nt 8633 to 8919) gave similar results. Hybridization with sense probes was negative. Apoptotic cells were detected by the TUNEL method, using In Situ Cell Death Detection Kit, Fluorescein (Roche), on frozen sections previously treated for *in situ *hybridization. After PBS washing, sections were incubated for 1 hour at room temperature with the TUNEL Label Mixture containing terminal transferase.

### Fadrozole treatment

White Leghorn eggs, were treated on day 4 of incubation (HH stage 24) with a single injection of 1 mg fadrozole (CGS 16949A), a specific nonsteroidal aromatase inhibitor (Novartis, Basel, Switzerland), as described [66]. Embryos were sacrificed at 14 days of incubation.

## Competing interests

The authors declare that they have no competing interests.

## Authors' contributions

DCE conceived the study, carried out the SSH selection and molecular biology studies, and drafted the manuscript. *In situ *hybridization studies were designed by SM and DCE, carried out by SM and NC and analyzed by SM who participated in drafting the manuscript. All authors read and approved the final manuscript

## Supplementary Material

Additional file 1 **Figure S2**. Effect of the internal polyadenylation signals. Click here for file

Additional file 2 **Figure S3**. Partial zebra finch *Ovex1 *sequence. Click here for file

Additional file 3 **Figure S4**. Alignment of chicken and zebra finch *Ovex1 *5'-proximal DNA sequences. Click here for file

Additional file 4 **Figure S5**. Alignment of Ovex1 putative proteins. Click here for file

Additional file 5 **Figure S6**. Structure and conservation of *ORF3*-encoded proteins. Click here for file

Additional file 6 **Table S7 – Polymorphisms in chicken *Ovex1 *sequences**. Polymorphisms observed in the *Ovex1 *sequence between the Red Jungle Fowl (genomic sequence galGal3) and the white Leghorn chicken strain (this study). Click here for file

Additional file 7 **Figure S8**. Alignment of Ovex1 RT and homologous sequences. Click here for file

Additional file 8 **Figure S9**. Detection of apoptotic cells in chicken ovary at hatching. Click here for file

Additional file 9 **Table S1**. Primers and PCR conditions. Click here for file
