# Supplementary material for: OVEX1, a novel chicken endogenous retrovirus with sex-specific and left-right asymmetrical expression in gonads
Source: Retrovirology. 2009 Jun 17;6:59. doi: 10.1186/1742-4690-6-59 (PMC2717909; doi:10.1186/1742-4690-6-59)
Supplement: Additional file 1 — Figure S2. Effect of the internal polyadenylation signals. [file 1742-4690-6-59-S1.pdf]

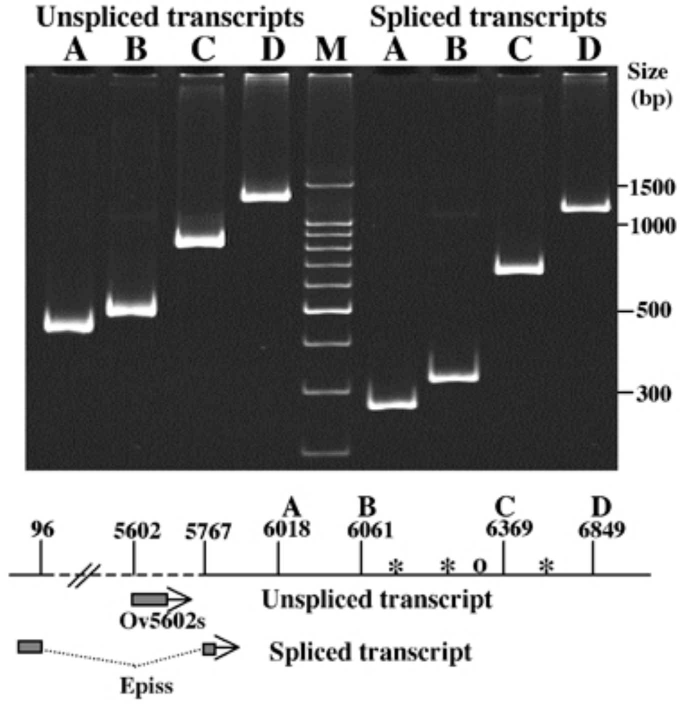

Figure S2. Effect of the internal polyadenylation signals

Ovex1 transcripts present in the adult chicken ovary were amplified by RT-PCR using either the sense primer Ov5602s, located into the intron, to amplify the unspliced mRNAs (left), or Episs, a primer overlapping the splice site to amplify specifically the spliced mRNAs (right). Antisense primers were Ov6018a in lanes A, Ov6061a in lanes B, Ov6369a in lanes C, and Ov6849a in lanes D. Sequences of primers are given in additional file 9 (Table S1, Primers and PCR conditions). The position of the AAUAAA polyadenylation site (nt 6280) is indicated by **o**, those of AUUAAA sites (6082, 6212 and 6664) by *. Molecular weight markers (100-bp DNA ladder) are in lane M. The result shows the presence of transcripts amplified with antisense primers located downstream from the polyadenylation signals (C and D). This indicates that transcription is able to override the internal polyadenylation signals in the case of the spliced and of the unspliced mRNAs.
