# Supplementary material for: OVEX1, a novel chicken endogenous retrovirus with sex-specific and left-right asymmetrical expression in gonads
Source: Retrovirology. 2009 Jun 17;6:59. doi: 10.1186/1742-4690-6-59 (PMC2717909; doi:10.1186/1742-4690-6-59)
Supplement: Additional file 2 — Figure S3. Partial zebra finch Ovex1 sequence. [file 1742-4690-6-59-S2.pdf]

```
                                                                                TATA box
                                                              - 40   ACTTGCCATGGCTATAAAAATCACCAGGCAGAGACTTC
                                                                                     SD
  +1 GCGGCCACCTTTACCTTAAAGGTGCTGGATCTTCTGGACCTCTGCAGAGACCATCTCCAAACTCCTGGGACTGCCGGAACGTTCAGCCGAGGATTG|GTGAGATCTTATTACTGGGGTCCT
 121 GCTCCATCTCTCCTTTGCCTCACCCTCTCCTGCATCCCTTCCCCGTTCCCTCCCGGGAGCCGGTGGCTTGCGGCAGCTGCCGGCTCGGCTGCGGCCTCCTGTTCCCACCTTGCCCTTCCC
 241 TTGTGCCCAGGCTCGGTGTCCCCCCGGGGGATTTTCGGAAGGGTGGGCTGTGGGGTTGCAGCCTCGCTCTGTGGAGGGGTTGAAGGGGCTGTCAGTGCTGGTGTGTTATGGTAGTTTCGC
                                                         Gag→
 361 CCTGAGACGGAGCCGTCTCAGATACTTGTCCCCTTGGCTATGATGAGCGACATCGTTAGCAGCTGGAAGCTGGAGCAGTTTGTGCTCCAGAAGTGTCTCCCCCCAATTTGGTCAGCGGGG
                                                             M  M  S  D  I  V  S  S  W  K  L  E  Q  F  V  L  Q  K  C  L  P  P  I  W  S  A  G     27
 481 TCTTTCCAGGGCCCGGATGCGTACCAGCAGCTGTGCCAGCAGTGGGAGAGCTGGTCCGAGGAGAACAAAAAGCCCAAACCCACTAACAAGTGTAAGAAGCGAGCGGCTTTGCAGGGTTTG
     S  F  Q  G  P  D  A  Y  Q  Q  L  C  Q  Q  W  E  S  W  S  E  E  N  K  K  P  K  P  T  N  K  C  K  K  R  A  A  L  Q  G  L     67
                                                                                 NLS
 601 CTGATGGTAGGCAGGGAGTTGTCCAAACTGCTGAAGGAGGCTCTTGAGAATGTGCAGACCTTGGAGCAGGCCAAGGAGGAGCTCAAAATCCAGGTGGACAACCTGCGGGCGGAGGTGCAG
     L  M  V  G  R  E  L  S  K  L  L  K  E  A  L  E  N  V  Q  T  L  E  Q  A  K  E  E  L  K  I  Q  V  D  N  L  R  A  E  V  Q    107
 721 GGCTTGTGTGGGGACTCTCTGCGGAGCGCGGTGGAGATCACCCGGCTGGAAAGCAAGCTGGGCTATGAGAAGCTGAAAACGGAGGAGCTGAAGAAGGAGGTTGGCAAGTGGATTGGGGAG
     G  L  C  G  D  S  L  R  S  A  V  E  I  T  R  L  E  S  K  L  G  Y  E  K  L  K  T  E  E  L  K  K  E  V  G  K  W  I  G  E    147
 841 ACCCACGATGCGCAGAGCGCGGTGCGGGCGGTCCTGCAGGACGTCCAGCGGGACCGGGGCACTGGCCCCGATCACAAGGTGTGCCATGCCAAGATCCAGGAGCTGGAGGCAGAGCTGGGG
     T  H  D  A  Q  S  A  V  R  A  V  L  Q  D  V  Q  R  D  R  G  T  G  P  D  H  K  V  C  H  A  K  I  Q  E  L  E  A  E  L  G    187
 961 GTGTCAAGGGGCATTGTGGCTGCCATTAAAGGCAAGAGGACCCCGATGGGGAATGGGGAGGGGGAGGACTCCCTGGACCTGCACCCACCCCACTACGATTATGAGGATGATGTGTGGGGT
     V  S  R  G  I  V  A  A  I  K  G  K  R  T  P  M  G  N  G  E  G  E  D  S  L  D  L  H  P  P  H  Y  D  Y  E  D  D  V  W  G    227
1081 GCTAACAGCCCCACCAGGCCGTCCCCCTATGCTCCTCTGCGGGAGGAGGTGTGCCAGCTGCAAGTAGGGGAGGTAGAGGCTTCCAAAGATAAAAAGGTTCCCCACGAAGAGCCAGTCAGC
     A  N  S  P  T  R  P  S  P  Y  A  P  L  R  E  E  V  C  Q  L  Q  V  G  E  V  E  A  S  K  D  K  K  V  P  H  E  E  P  V  S    267
1201 CATGCCCCACTGCAGCCCATAGACACCAACAGGGCTGTGCTCTGGTTTACCCCTGAGCAGATTAAGACTGTGGGGAAGATGCTGGGGCCGCTGACAAAGGAGACAGCAATTAACTGGCTG
     H  A  P  L  Q  P  I  D  T  N  R  A  V  L  W  F  T  P  E  Q  I  K  T  V  G  K  M  L  G  P  L  T  K  E  T  A  I  N  W  L    307
1321 TCCAGGGTCCAGCGTCTGCCCAAGTGCCAGAGTGGCAACTTTGTGAGTGACCTGATAGACATTGTGAGAAAGTGCATGAAACCAGATGATTTTGCGTCACTGCCAGGGGATGTGCAGATG
     S  R  V  Q  R  L  P  K  C  Q  S  G  N  F  V  S  D  L  I  D  I  V  R  K  C  M  K  P  D  D  F  A  S  L  P  G  D  V  Q  M    347
1441 GGCAATGTCCAGGATATCGGGGATGTCCAGCTGGCTGTGCTGAAGGTGTTCTTCCCAGAGGTCAACCCCTTAGTGCTCTTCCACCAGGAGAAGCAAAACCCCGAGGAGAGGCCTGATGCC
     G  N  V  Q  D  I  G  D  V  Q  L  A  V  L  K  V  F  F  P  E  V  N  P  L  V  L  F  H  Q  E  K  Q  N  P  E  E  R  P  D  A    387
                                                                                                      MHR
1561 TATGTTAACCGTAAAAGGATGCTCTACCAGATGGCTGGGCTGCCTGGCTCAAAGGAGTCCCCCCCTGATTTTGACAGGCCTGAATTCAAGGAGCCACTGGTGGTGGGGTTGACACCCCCA
     Y  V  N  R  K  R  M  L  Y  Q  M  A  G  L  P  G  S  K  E  S  P  P  D  F  D  R  P  E  F  K  E  P  L  V  V  G  L  T  P  P    427
1681 CTGCGGGTGATTGCTGGTGGGGATGCAGCCAAAAAGCCTCTCTCAGAGCTGGAGCAGATCCTGAACCAGAATTTTGAGCTGCAAAAGCAGGCATTCCCGGGATACATGCTGGGGGGGAAG
     L  R  V  I  A  G  G  D  A  A  K  K  P  L  S  E  L  E  Q  I  L  N  Q  N  F  E  L  Q  K  Q  A  F  P  G  Y  M  L  G  G  K    467
1801 AAAGGGAAAGCCTTGGGCATGTCTTTCCAAAATGCAGGGATGAGAAAAATGCAAGGAGACAACAGAAAAGATCCTGATAGCAAGGGGGGGTTGGGGAAGGAGAACCAGCAAGGGATGAGG
     K  G  K  A  L  G  M  S  F  Q  N  A  G  M  R  K  M  Q  G  D  N  R  K  D  P  D  S  K  G  G  L  G  K  E  N  Q  Q  G  M  R    507
1921 TTTCAGAAGTCTAACCCTTGGCGGGCTGAGCTGAGGAAGCGCTTGATAAAATATGAGAAACAGGAGGACATTGATGGCTTGCCGGATGCGGAGCTCTTCCAAAAACTGGCCCTGCATGAG
     F  Q  K  S  N  P  W  R  A  E  L  R  K  R  L  I  K  Y  E  K  Q  E  D  I  D  G  L  P  D  A  E  L  F  Q  K  L  A  L  H  E    547
                                                Pro-Pol→
2041 GCCAAAAAAAACAGCAGCTCCCACCTGATTGACCCGGGGGCTAAGGCTCAGCAATAGGGCTGCCAGGCACCCGCATCGCCCCTTTTTGTGGCACCGATTAAGACCGATTTGTGGGGCGC
     A  K  K  N  S  S  S  H  L  I  D  P  G  A  K  A  Q  Q  *  G  C  Q  A  P  A  S  P  L  F  V  A  P  I  K  T  D  L  W  G  R    587 |
2161 CTGATAGTTGATACAACTATTGGAGGAGGGGTGCTTGGACAAGTGATGTTAATTGATACTGGTGCCTCTTATTCAATTCTGAATATGGCCCCCGGCAGAGCCGGGATCTTCCAAACCTCT      |
     L  I  V  D  T  T  I  G  G  G  V  L  G  Q  V  M  L  I  D  T  G  A  S  Y  S  I  L  N  M  A  P  G  R  A  G  I  F  Q  T  S    627 | Protease
2281 GAAATTATTGAACTGACAGGGCCGAACGGCCAGAAATCCTCAGTGCCGTTCACAGGGCCCATACCCTTTGAAATTGGGGCTGCCAAAGGGTCTGAAAAATTTGGGAAAATAGAAATGAAA      |
     E  I  I  E  L  T  G  P  N  G  Q  K  S  S  V  P  F  T  G  P  I  P  F  E  I  G  A  A  K  G  S  E  K  F  G  K  I  E  M  K    667 |
2401 GGAAAGCCAGGAGTTTTAGCTATCTCTGCACTGACAAAGATGGGAGTGGTGGTGGACCTGGCAAACCAAGCATTGCTACACTGCCCACAAATTGATTTGCCCGTCATCCCGGCAAGCCAC      |
     G  K  P  G  V  L  A  I  S  A  L  T  K  M  G  V  V  V  D  L  A  N  Q  A  L  L  H  C  P  Q  I  D  L  P  V  I  P  A  S  H    707 |
2521 AGGGTGATGTCAGTGAAAGCCCCTGAGATCCTGGTCCACCCGGAGTGGGAGCCCCGGGTGAAACGGGTCACAGAGCAGTTCCCCCAGGTCTGGGCAAGGAGCAAGCTGGACTGTGGGCAG
     R  V  M  S  V  K  A  P  E  I  L  V  H  P  E  W  E  P  R  V  K  R  V  T  E  Q  F  P  Q  V  W  A  R  S  K  L  D  C  G  Q    747
2641 ATTGATGCTGCTGTGTCAATCAAAGGGCCAGATCCGCCTCCTCAGGCTCAGCCCTGGTACCCGGCTGAGGCTGAGGACAGCTTGTGGGACACGGTCAAAACCTTGCTGGACCAGGGCATG      |
     I  D  A  A  V  S  I  K  G  P  D  P  P  P  Q  A  Q  P  W  Y  P  A  E  A  E  D  S  L  W  D  T  V  K  T  L  L  D  Q  G  M    787 |
2761 CTGGTGGAGCAGCAGAGCACCAACAATGCCCCAGTGTGGCCCCTGAGGAAAGCTGATGGGAAAATGTGGAAGCTGACAGTCGATTTCAGTGCCCTGAACCAAGTCACCCCAATGCAGACC      |
     L  V  E  Q  Q  S  T  N  N  A  P  V  W  P  L  R  K  A  D  G  K  M  W  K  L  T  V  D  F  S  A  L  N  Q  V  T  P  M  Q  T    827 |
2881 CCCACGGTGGTCAGGTATCCCAACATCATGGCAGCCATCTCCCGGGGCTCCGAGTGGTTCTCGGTGCTCAGCCTGACCAGCCCATCATTTGCTGTCCCTTTGCACCCTGACTCCTGGCAC      |
     P  T  V  V  R  Y  P  N  I  M  A  A  I  S  R  G  S  E  W  F  S  V  L  S  L  T  S  P  S  F  A  V  P  L  H  P  D  S  W  H    867 | RT
3001 AAGTTTGCCTTCACGATTCGAGGACGGCAATTTGCTTTCACCAGAGTCCCTCCGGGTTTCCACAGCGCCCCTGCTATCTGCCATGCCCGCGTGGTGAGGATGTGGGAGCAGGTCAGCCAC      |
     K  F  A  F  T  I  R  G  R  Q  F  A  F  T  R  V  P  P  G  F  H  S  A  P  A  I  C  H  A  R  V  V  R  M  W  E  K  V  S  H    907 |
3121 CGGGAGAGCGTGCTGTCCTGTGCTGGTGACATCCTCATCCACACCCAGACAAAGGAGAAGAACCTGGAGGTGCTAGCAGAGGTGCTGGGGGCTATCCAGAAGACAGGCTTCAGGATCAGC      |
     R  E  S  V  L  S  C  A  G  D  I  L  I  H  T  Q  T  K  E  K  N  L  E  V  L  A  E  V  L  G  A  I  Q  K  T  G  F  R  I  S    947 |
3241 CCCACAAAGGCCCAGCTGTGCTGGAAGGAGGTCTCCTTTGTGGGGGTGACCCTGGGGGAGGAAGGGCGCTCACTGGAAAAGCAGAGGGTTGAGCTAATACAGAAGATGTCAGCACCATCT      |
     P  T  K  A  Q  L  C  W  K  E  V  S  F  V  G  V  T  L  G  E  E  G  R  S  L  E  K  Q  R  V  E  L  I  Q  K  M  S  A  P  S    987 |
3361 GATACCACCACCCTGAGGTCCTTCCTGTTCCTCCTGAGGTTGTACCGTGAGTTCATTGAGGGCTATGCAGAGAAAGCAGCCCCACTTTGCCACCTCCTGAAAAAGAACACCCCCTGGGAA      :
     D  T  T  T  L  R  S  F  L  F  L  L  R  L  Y  R  E  F  I  E  G  Y  A  E  K  A  A  P  L  C  H  L  L  K  K  N  T  P  W  E   1027 :
3481 TGGGGGCCAGAGCAGGATGAGGCAGTGAAAACCTTAAAGGAGAGTGTGGCACGAGCCCCCATGCTGGCACATCCTCAAGGGGACAGACCCTACGTTCTGCAGCTGGCCAGCACGGGGATG      :
     W  G  P  E  Q  D  E  A  V  K  T  L  K  E  S  V  A  R  A  P  M  L  A  H  P  Q  G  D  R  P  Y  V  L  Q  L  A  S  T  G  M   1067 :
3601 GGGCTGCAGGCCACCCTGGGCCAGCAGCACAGTGCCAAGCTGCTCCCTGTTGCCCACGCCTCGCGTCTCCTGACGCCAGCCGAGCAACAGTTCAGTGACTACGAGAGGGAGGTTTTAACC      : Tether
     G  L  Q  A  T  L  G  Q  Q  H  S  A  K  L  L  P  V  A  H  A  S  R  L  L  T  P  A  E  Q  Q  F  S  D  Y  E  R  E  V  L  T   1107 :
3721 CTAACCTGGGCCCTGCAGCACTGGGAGTAC--- 3750                                                                                     :
     L  T  W  A  L  Q  H  W  E  Y  ---    1117                          Gap

4420 ---GAAGCCAAGTGGAACAAGGAGGCAGATGCCAAAGCCAAGCAAGGTGCTAAGGAAGGGCAGCTGTGGAAAGCCAGCAAGATGTCAGAGAGTGGCCCAGTGCTGGTGGCTCCCAGCAGG        RnaseH
  1341-E  A  K  W  N  K  E  A  D  A  K  A  K  Q  G  A  K  E  G  Q  L  W  K  A  S  K  M  S  E  S  G  P  V  L  V  A  P  S  R   1379 |
4537 CACTGGGAGAGTGTGGATCTGGTGGGCTTGCAGGCACAGGACCCCAGGCTCTGGGAGTTAGCACAGGGCAGGGAGTACAAAGGGTGCAGGGTGTGGAAAGATGTCTCAGGGCTGATCCTG      |
     H  W  E  S  V  D  L  V  G  L  Q  A  Q  D  P  R  L  W  E  L  A  Q  G  R  E  Y  K  G  C  R  V  W  K  D  V  S  G  L  I  L   1419
4657 GCACGGAGGGAGGGGGCAGATTTCCCGGTCTGGGTGGTGCCTGAACTGGTCCGGACAGAGCTGGTGGCCCTGGCTCATGAGCAGGGGCATCTGGGGACAGACAAGACCATGGTGAGGCTG      |
     A  R  R  E  G  A  D  F  P  V  W  V  V  P  E  L  V  R  T  E  L  V  A  L  A  H  E  Q  G  H  L  G  T  D  K  T  M  V  R  L   1459 |
4777 CAGGGTGCTGGGTGGTGGCCCGAAATGAGGGAGGATGTGGAGAGGTATGTCAGCAACTGCCTGACATGTGCAGCCAACAATCCTGATGCCAAAGTAGCCAAAGCCATCCTGGGCTACCA       |
     Q  G  A  G  W  W  P  E  M  R  E  D  V  E  R  Y  V  S  N  C  L  T  C  A  A  N  N  P  D  A  K  V  A  K  A  I  L  G  Y  Q   1499 |
4897 AGGGTTTCTGGGCCATGGAGCAAGTTGCAGATGGAGTTTATTGGCCCTCTGTCCCAAACAGCCCAAGGCAATAAGTACTGCCTAGTGGTCAGTGATATCTTCACTAAATGGGTGGAAGCA      |
     R  V  S  G  P  W  S  K  L  Q  M  E  F  I  G  P  L  S  Q  T  A  Q  G  N  K  Y  C  L  V  V  S  D  I  F  T  K  W  V  E  A   1539 | Integrase
5017 TTCCCAGCTCGAAACAACTCAGCCAGTGCAACTGCAAAGATCCTGGTAGAGCACATCTTCTCACAGTGGGGCATCCCAAAGGAGATTCACTCAGAGCACGGCCAACGCTTCATTGGGGAA      |
     F  P  A  R  N  N  S  A  S  A  T  A  K  I  L  V  E  H  I  F  S  Q  W  G  I  P  K  E  I  H  S  E  H  G  Q  R  F  I  G  E   1579 |
5137 GTCACAAAAGGGGTGTGCCAGGCCCTGGGAATCAAGCAGAAGCTCCACATCACAGGGCATTTCCAGAAGCCAATCTCAGCAGAGGGGCCCAACCAACCACTGAAGACAGCCCTGAAGAAA      |
     V  T  K  G  V  C  Q  A  L  G  I  K  Q  K  L  H  I  T  G  H  F  Q  K  P  I  S  A  E  G  P  N  Q  P  L  K  T  A  L  K  K   1619 |
```

Figure S3

```
5257 CTTGTGAACCAGCAAAGGAAAGACTGGGATCATAAGCTCCCACTGACCTTGCTGGCATTGAGGGGGTCTCTGGCATCTCAAACACTTTCTCCCCCAAAATTTCCTCCCTCAAGGGACCCG
      L  V  N  Q  Q  R  K  D  W  D  H  K  L  P  L  T  L  L  A  L  R  G  S  L  A  S  Q  T  L  S  P  P  K  F  P  P  S  R  D  P  1659
5377 AAGTTTCTGGAACACTGGTGGCAAGGAGGGGAGCCCCCAGATGAAATGCAGCCCCGCGTGCTGATGGACAGGTGGGTCCAGGACATGCTAAGGACGGTGTCAGCCACCTACCACCAGCTC
      K  F  L  E  H  W  W  Q  G  G  E  P  P  D  E  M  Q  P  R  V  L  M  D  R  W  V  Q  D  M  L  R  T  V  S  A  T  Y  H  Q  L  1699 Integrase
5497 GCCTCAGTGCAGGAGACCAGCATCCCTAAGATGGATAAGCAGTTGGGAGTGCTACTGCGCCCCATGGAGTGGAACACAGGGGACCTGGTGATATACCGAGGGGTTAGGGAGAAGAACCAG
      A  S  V  Q  E  T  S  I  P  K  M  D  K  Q  L  G  V  L  L  R  P  M  E  W  N  T  G  D  L  V  I  Y  R  G  V  R  E  K  N  Q  1739
5617 GTGCTGGAACCCCAGTGGATGGGGCCTGTTAGGGTTGTGAACAAGGCCAGCCCCTCTGTGTACCAGGTAGAAATCAGAAAAGGGGCAAAAAGGCAGGAGAAATGGTTCCATTCTTCACAA
      V  L  E  P  Q  W  M  G  P  V  R  V  V  N  K  A  S  P  S  V  Y  Q  V  E  I  R  K  G  A  K  R  Q  E  K  W  F  H  S  S  Q  1779
                                                                     SA          ORF3→
5737 TTAAAAGCATGGAAGGGAAACTAACGGGGCTGGAGAGCCCGCCTTGCTTATGTTTTGCAGATAAAGGAAAATGGAAGGCGATTGTGACCAGCGGGAAGGGCGATCTTCGTGGCATCACCA
      L  K  A  W  K  G  N  *     1786                                    M  E  G  D  C  D  Q  R  E  G  R  S  S  W  H  H  Q    17
5857 GCCCTGTTTGTGCTGTGGGGAAAATGCTTTCAAATTTGTGATTGCAGGATTTACTTTGCTTTCTGGTATGTGCATAGTGCTGAGCACCCAGTGCGTCCAACCAACCCATCAGCATCTTGG
      P  C  L  C  C  G  E  N  A  F  K  F  V  I  A  G  F  T  L  L  S  G  M  C  I  V  L  S  T  Q  C  V  Q  P  T  H  Q  H  L  G    57
5977 TAAAAATGTCATTCATTCTCACTTAAAAAGGCACCTTGACACACAGGCCACAGCCCAGCACAGGCTTAGGAGGCACACAAAAATTGGGACTGACTGGCCCTGGTCCCAAGCATACATAAA
      K  N  V  I  H  S  H  L  K  R  H  L  D  T  Q  A  T  A  Q  H  R  L  R↑ R  H  T  K  I  G  T  D  W  P  W  S  Q  A  Y  I  K    97
6097 ACACACAGGGATCATGGGATTAAATTCTAACAAAGGCTTAAATTTGTCAACAGTGGTTATGCATGGGTCAGAGGTGTACTTGGAAAATGAGTGGGGCTGGGACAGCACAAACAGACTTCC
      H  T  G  I  M  G  L  N  S  N  K  G  L  N  L  S  T  V  V  M  H  G  S  E  V  Y  L  E  N  E  W  G  W  D  S  T  N  R  L  P   137
6217 ACAGCTGCTGGGAAAGGTGGGACAGCAAATAAAGGTGGGGTGCAGGGTAATTAACGGATCCACTCACCAGCGAGTAACTCAAATCTCTGTCACTGAAATAAAAACTAAGAAGAATCAGAA
      Q  L  L  G  K  V  G  Q  Q  I  K  V  G  C  R  V  I  N  G  S  T  H  Q  R  V  T  Q  I  S  V  T  E  I  K  T  K  K  N  Q  N   177
6337 CAAAATCTGTGCTGTGGAGAACATGGACTGCTGGTTCAATTTTACTTTGGTCCAGCCAGTCTTTGTGGTTTGCCTCTGGGCACAAAACAGTGTGGGGCTGTCTTTTAAATTTAAGATCAG
      K  I  C  A  V  E  N  M  D  C  W  F  N  F  T  L  V  Q  P  V  F  V  V  C  L  W  A  Q  N  S  V  G  L  S  F  K  F  K  I  S   217
6457 CACCACAGCACCCTCCTTTGCTGCCATTAAGATCTCAAAGTGCCATTTTTTGGCCTGGCATGCACCCCGTTATGTTGAAATTGGCAACCAGGTAAAAGTGGAAATCAAATACTCCCAAGA
      T  T  A  P  S  F  A  A  I  K  I  S  K  C  H  F  L  A  W  H  A  P  R  Y  V  E  I  G  N  Q  V  K  V  E  I  K  Y  S  Q  E   257
6577 AAATGTAGCTGACCCAGTGCAGATTAATGGCACCACTGTGACTGTCACAGCCCCCAACAGCCGCAAGTGGATCATTCCACTGACCTGCCTCTGTGAGACCAAAACACTTGACAGATCTGA
      N  V  A  D  P  V  Q  I  N  G  T  T  V  T  V  T  A  P  N  S  R  K  W  I  I  P  L  T  C  L  C  E  T  K  T  L  D  R  S  E   297
6697 ATTAGATGCAGAGACTTCATGGTATAATCAGGATTATGGACGCTGCCCACACCTGATCATAACCCTCCAGGTGTGGTGTCAAGGAAACCTGAGCGTAGAAATGTCTCCCAAACTTGGAGG
      L  D  A  E  T  S  W  Y  N  Q  D  Y  G  R  C  P  H  L  I  I  T  L  Q  V  W  C  Q  G  N  L  S  V  E  M  S  P  K  L  G  G   337
6817 AAAGTGGTGGATAGGAGGCCCTGAAGGATTTAAAAAAGAATTCACCATCACTGCTGTCTTGCCCCCTGTTGTTTCCAAAATAGGTCCTTATGTAGTGAAGAAAAATCACATCCAGGAGCT
      K  W  W  I  G  G  P  E  G  F  K  K  E  F  T  I  T  A  V  L  P  P  V  V  S  K  I  G  P  Y  V  V  K  K  N  H  I  Q  E  L   377
6937 GTTGACAGGGCCTGTCCGGTCACTAAAGAAGGTGGTGCTGTCTCTCTCCACTGTTAACATTTCCTCTATCAGACCACACTGTGCTCCCTTCCTGTCTACTCTGCACACTGGCTGGCTGGC
      L  T  G  P  V  R  S  L  K  K  V  V  L  S  L  S  T  V  N  I  S  S  I  R  P  H  C  A  P  F  L  S  T  L  H  T  G  W  L  A   417
7057 ATGGCTCCACAGCCGCTCCCTCCAGGGCACCCGGGCCAGGAGGGACCTGCTGGCCACAGCGCTGGGAGGAGGAGCTGCTGGCCTGGGAGTCCTCAACAGCATGGATGCTGAAGTCTTGGC
      W  L  H  S  R  S  L  Q  G  T  R  A  R↑ R  D  L  L  A  T  A  L  G  G  G  A  A  G  L  G  V  L  N  S  M  D  A  E  V  L  A   457
7177 AAACAAGCTGGAGACTGTCACCTTGGGCGTGCAGGACCTCCTCAAGCCCCTGAATTCATCCCTGTCCAGCCTTGGGATGGGGCAGTGGCTTGTGTCAGAGGTGCTGCCCACCTGGGAACA
      N  K  L  E  T  V  T  L  G  V  Q  D  L  L  K  P  L  N  S  S  L  S  S  L  G  M  G  Q  W  L  V  S  E  V  L  P  T  W  E  Q   497
7297 AATAAGTGAGAAAGACCATCAGGTGCTGTTGCGAGCCCTGGGCATGGAACAAAGTAACGTCTCGCTTGCTCTTAGCTGCATCCAGGCCCAGATGTGGGTGCAGAGTGTTGTTGCAGGTAT
      I  S  E  K  D  H  Q  V  L  L  R  A  L  G  M  E  Q  S  N  V  S  L  A  L  S  C  I  Q  A  Q  M  W  V  Q  S  V  V  A  G  I   537
7417 CCTGAGAGATGGTGACAACGGCGTCCTGCCCACTGAGATCCGAAAGATCGTGTGGGACGCGGCCACAGAGAAGGAGCGGCAGCTCCAAGCCTGGTGGAGGCTCGTCAACTTCACCCACGA
      L  R  D  G  D  N  G  V  L  P  T  E  I  R  K  I  V  W  D  A  A  T  E  K  E  R  Q  L  Q  A  W  W  R  L  V  N  F  T  H  D   577
7537 CCAGGCCCTCAACGCGGTCGTTGCCCACGTCCTCACTGTGGCGGAGGCTCGCATTGAAAAGGTCTACCCCATCGTGGCCCTGGGAGTAAACACAAATGGCTCTGTGGTCTACCCCCTGGA
      Q  A  L  N  A  V  V  A  H  V  L  T  V  A  E  A  R  I  E  K  V  Y  P  I  V  A  L  G  V  N  T  N  G  S  V  V  Y  P  L  D   617
7657 CCACCGCATGTGGGCCAGGGTGTCTGAGGGGAGGTGGGAGACGGTAGATTTGGACGCCTGCATTTTGGAGAGGGGGCTGGGATTCATATGCGAGGATGATGCCCTTAAGGCGAGCGATGT
      H  R  M  W  A  R  V  S  E  G  R  W  E  T  V  D  L  D  A  C  I  L  E  R  G  L  G  F  I  C  E  D  D  A  L  K  A  S  D  V   657
7777 GTGCTTTGACACCAAAGAAGGGGTGTGCCACTTTGAGATCAACCCCCAGAGCAGTAACAAAACCATGTTAGTGTACGTAGGGAAAGGCTGCGTGTGCTTCAGAACGGCGTGTAAATACGT
      C  F  D  T  K  E  G  V  C  H  F  E  I  N  P  Q  S  S  N  K  T  M  L  V  Y  V  G  K  G  C  V  C  F  R  T  A  C  K  Y  V   697
7897 GCAAATTAATGAAGCTTATAACCAGACTGTGTTTAGCGACTCAAATATGTGTGCTTGCAATGTAGCTACTATTAGAGGCTGTGATTTTGTGTATAAACCACCTGTGTTTACTAGCCAGTT
      Q  I  N  E  A  Y  N  Q  T  V  F  S  D  S  N  M  C  A  C  N  V  A  T  I  R  G  C  D  F  V  Y  K  P  P  V  F  T  S  Q  L   737
8017 GCTAATCAGAAACTATACCTTGTATCGTAGTATAACCCCCACCCCCATCGGTATGGATTTATCACTTGTAAAAGAAATGTTAGAGCATGCAAATTTGCAGCAGCTGCTAGAAAATGCCAA
      L  I  R  N  Y  T  L  Y  R  S  I  T  P  T  P  I  G  M  D  L  S  L  V  K  E  M  L  E  H  A  N  L  Q  Q  L  L  E  N  A  K   777
8137 GGCTGAAGCTAAAAAGATCCTAATAACGGTTCACCATGATGGTAAAGTTATAAAACAGGTAATGGAACGAATTAAGAGGGCGGGAGAACACCACTGGTGGGAGGCTTTTTTTGGCTGGTC
      A  E  A  K  K  I  L  I  T  V  H  H  D  G  K  V  I  K  Q  V  M  E  R  I  K↑ R  A  G  E  H  H  W  W  E  A  F  F  G  W  S   817
8257 CCCCACGGCCACTGGCATTTTCAACGCGCTGCTGCACCCCATTGTAGTTATCCTGCTAATGCAAATCTGTGTGTGCTTTGCCATGGTAGCCACTTGTTACCGGATGAGGCAGGTGAAGCT
      P  T  A  T  G  I  F  N  A  L  L  H  P  I  V  V  I  L  L  M  Q  I  C  V  C  F  A  M  V  A  T  C  Y  R  M  R  Q  V  K  L   857
                                                    Transmembrane domain
8377 CTGCTTTGATAACCAGATGAAAGAAGCAGGGCTGGCCAAGAGCCTCCTGAAATAGTCAAGGGCAAGACTGGGTTGGCCTCTGTGTTTGCCACCAAGAAGATCTTTTGGCTCCGCTGTTGG
      C  F  D  N  Q  M  K  E  A  G  L  A  K  S  L  L  K  *                                                                   874
8497 CTGCAACCCAGTAGGCGTTGAGCGGTGGGGACACACGCCATGCCAGACCTTGATGAGAGGGATGGCCTTCTCTGTCACCAGAGGGCATCCAGGAGGGGGAGGACAGGCCAAGCAGCCTGCA
8617 GATGGCATGAGTCTCAGAATGGCTGAGGTTGGAAGGGACCTCTTGAGAGGTTTCGTTTAGTCTGAACCCACCTGTTCAAGGAGGGTCACCTAGAGCAGATTGCCCAGGACTGTGCTCAGC CR1-Y4
8737 TGGGCTTTGACCATCTCCATGGATGGAGACTCCACAACTTCTCTGGGCAAACCTGTTTCATTGTTTGATCACCCTCATAGTAAAGAAGCAAAAGTTAGCATAAGTAGCTTGTTTTTGAAC
8857 TTCCCATCCCCAAATACTGATGTTAAGAAGCTGCTCTTGTGTGATTTAAGAGTCCTTAAACCAGGGAGATGGGGGTAAGGAAAGCCGAGGACACTGTCTTTGGAGGGAAAGAGAAGGAAA
8977 CGTGCTTGGAAAACACCCACTGAAGACAGCGGGGGTGTGAACAGACTCAAAGATTTGGAGAGATGGTAAAAGATGAGCAGAACAGAAGACCTTGTAATCACCATGGACTTTAAGGAGCTG
9097 AATCCACATGGACCTTTGCAGCATCACCTCCAGGTTGCCAAGACTCCCAGCACGTCTGGCCAGCGGATTGGTGAGATCTTGTTTTTGGAGCCTGGACATCACTGCTTTGCTTTGCTTTTC
9217 TTAGTTGTTCATTACTCAATAAATCTGTCTGTTAGAGAAT                 9256
                 Polyadenylation signal
```

Figure S3 – Partial zebra finch *Ovex1* sequence

Partial nucleotide sequence of the zebra finch *Ovex1* locus and predicted translation products. The nucleotide sequence is taken from the July 2008 *Taeniopygia guttata* genome draft assembly (taeGut1) (chr. 4 (+) 65,939,815 to 65,948,592). Nucleotides are numbered on the left, according to the alignment with chicken *Ovex1*. The finch DNA sequence gap is assumed to contain the same number of nucleotides as the corresponding sequence of the chicken. The 5' and 3' imperfect direct repeats are indicated by dashed underlines. Sequences identified as similar to GGLTR11 and CR1-Y4 by RepeatMasker are on a gray background. The TATA box and polyadenylation signals (AATAAA and ATTAAA) are underlined. SD and SA indicate the putative splice donor and acceptor sites. The conceptual translation of *Gag*, *Pol* and *ORF3* is given under the nucleotide sequence, and amino-acid positions indicated on the right. Putative translation start codons are boxed. Conserved motifs are underlined: the Gag nuclear localization signals (NLS), leucine zipper motif and major homology region (MHR). In Pro, the active site is underlined. In RT, position of the usually conserved aspartate residues is indicated in bold underlined characters. In the integrase, amino acids involved in the zinc-binding finger are in bold characters and residues of the degenerated DD35E motif bold and underlined. In the ORF3 protein, potential N-glycosylation sites are in bold letters, potential cleavage sites indicated by arrows, and the transmembrane domain underlined. In the 3'-UTR, the 19-base polypurine tract is doubly underlined.
