# Supplementary material for: OVEX1, a novel chicken endogenous retrovirus with sex-specific and left-right asymmetrical expression in gonads
Source: Retrovirology. 2009 Jun 17;6:59. doi: 10.1186/1742-4690-6-59 (PMC2717909; doi:10.1186/1742-4690-6-59)
Supplement: Additional file 3 — Figure S4. Alignment of chicken and zebra finch Ovex1 5'-proximal DNA sequences. [file 1742-4690-6-59-S3.pdf]

Figure S4 – Alignment of chicken and zebra finch *Ovex1* 5’-proximal DNA sequences

```
                        Pitx2
Chicken       -211    ACTAATCCGGCCCCCCTAAAAAAAAAATAATGAGCAGAGCACCTGCTCCATGGTTATC -155
Zebra finch   -220    CCTAATCCTGGCCCTCCCCAAACAGA-TAAGGGGCAGTGCAGGTGCTACTGTGTGATC -165
                       ******* * *** *   *** * * *** * **** ***  **** *   ** ***

                                               GATA                   ERR
Chicken             GGTGC-----------------CAGAGGATAAAA------AGACAAATTGTCCTTGGGAA -119
Zebra finch         TCAGCTTCCCTAATCAGTGTTGAAGAGGATAAGGAAAGACAAGGAAAGTGTCCTTGGGAA -105
                       **                  *********      * *   *** ************

                                                                Forkhead
Chicken             GATAGCAGTGGAGATGCTGCACCTGAGGGAGGGAAAGGCACTGAGGAAGCAAACA--GGA  -61
Zebra finch         GGCA--------------GCACACGGAGGAGGAAAAGCCGCCAGGGAAGTAAACACAGGT  -59
                    *  *              ****  *  ***** **** * *   ***** *****  ** *

                                 Ras                 TATA
Chicken             GATATCATCCCCAAACAACGACTTGCCATGGCTATAAAGAGCTGCCGGGTCTGAGATCCT   -1
Zebra finch         GTCATCAGCCCCAAACAAAGACTTGCCATGGCTATAAAAATC-ACCAGG-CAGAGACTTC   -1
                    *  **** ********** ******************* *  ** ** * ****
```

Sequences from the genome draft assemblies galGal3 and taeGut1 were aligned with ClustalW2. Conserved nucleotides are indicated by *. Conserved putative responsive elements are enlightened: TATA box (TATA) and sites for Ras-responsive element binding proteins (Ras), forkhead domain factors (Forkhead), estrogen-related receptors (ERR), GATA binding factors (GATA) and bicoid-like homeodomain transcription factors (Pitx2). Arrows indicate the orientation of the elements.
