# Supplementary material for: OVEX1, a novel chicken endogenous retrovirus with sex-specific and left-right asymmetrical expression in gonads
Source: Retrovirology. 2009 Jun 17;6:59. doi: 10.1186/1742-4690-6-59 (PMC2717909; doi:10.1186/1742-4690-6-59)
Supplement: Additional file 4 — Figure S5. Alignment of Ovex1 putative proteins. [file 1742-4690-6-59-S4.pdf]

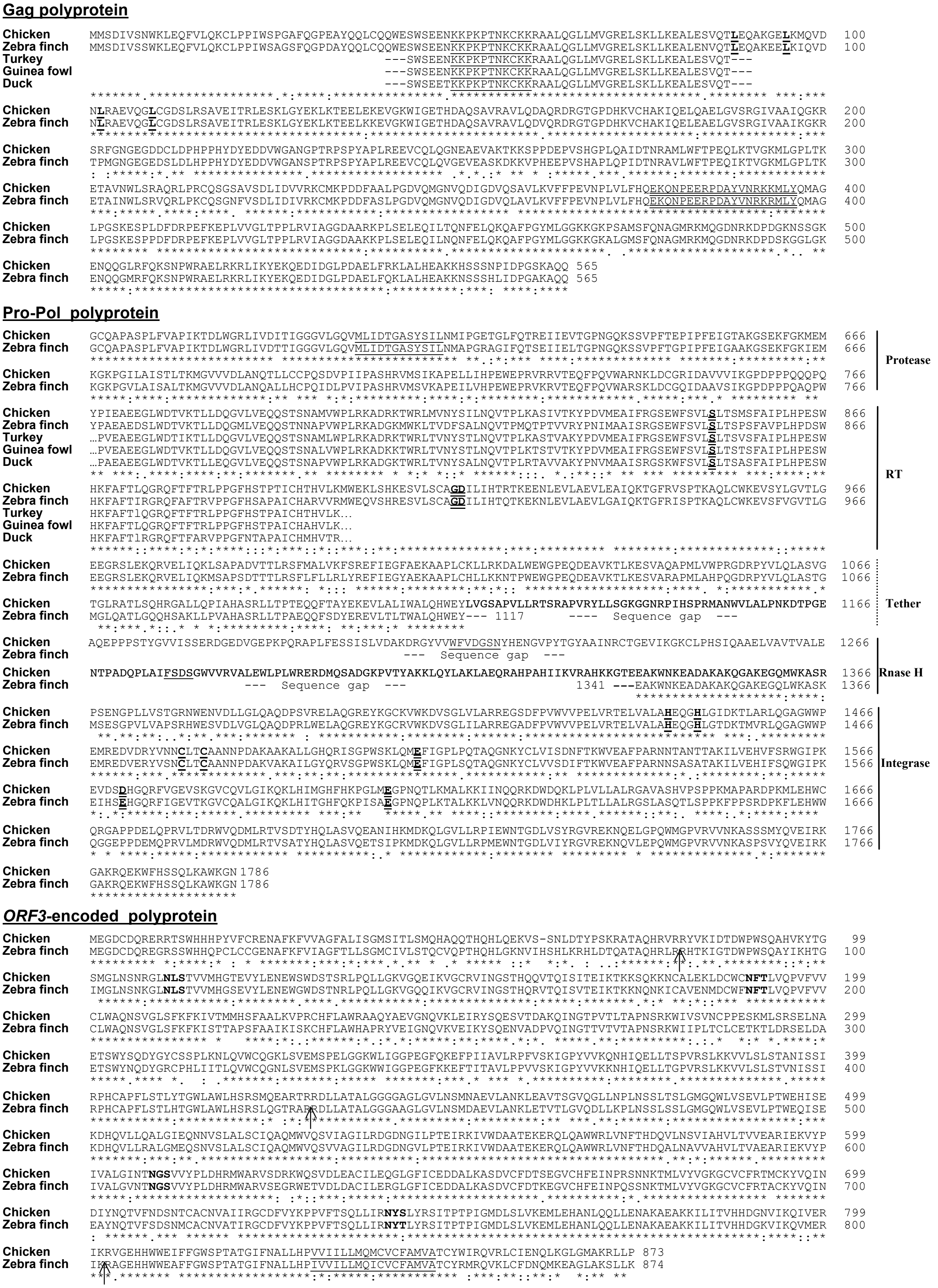

Figure S5 – Alignment of Ovex1 putative proteins

Alignment of chicken, zebra finch, turkey, guinea fowl and duck Ovex1 putative proteins. Protein sequences were aligned with ClustalW2. Conserved residues are indicated by * and similarity by (:) or (.). In Gag polyproteins, the nuclear localization signal, leucine zipper and major homology region are underlined. In Pro, the active site is underlined. In RT, position of the usually conserved aspartate residues is indicated in bold characters. In integrase, residues involved in the zinc-binding finger and in the degenerated DD35E motif are underlined. In the ORF3 protein, conserved putative cleavage sites are indicated by arrows, conserved putative N-glycosylation sites are in bold characters and TM domains underlined.
