# Supplementary material for: OVEX1, a novel chicken endogenous retrovirus with sex-specific and left-right asymmetrical expression in gonads
Source: Retrovirology. 2009 Jun 17;6:59. doi: 10.1186/1742-4690-6-59 (PMC2717909; doi:10.1186/1742-4690-6-59)
Supplement: Additional file 5 — Figure S6. Structure and conservation of ORF3-encoded proteins. [file 1742-4690-6-59-S5.pdf]

Figure S6 – Structure and conservation of *ORF3*-encoded proteins

A

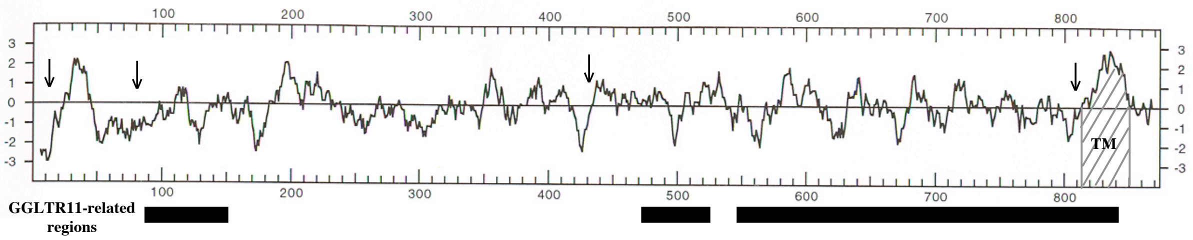

B

```
                 86                                                           151
Chicken      …TDWPWSQAHVKYTGSMG--LNSNRGLNLSTVVMHGTEVYLENEWSWDSTSRLPQLLGKVGQEIKVGCR …(320 aa)…
Zebra finch  …TDWPWSQAYIKHTGIMG--LNSNKGLNLSTVVMHGSEVYLENEWGWDSTNRLPQLLGKVGQQIKVGCR …(320 aa)…
GgLTR11      …TGWSRSHAHIGHTGRMGPNSNSNRGLTLSTTVKHSNEIYSGNEWGQDSTGRLPQLXGDAGRRIKVGCR …(10-11 aa/

              472                                                                           551
Chicken       PLNSSLTSLGMGQWLVSEVLPTWEHISEKDHQVLLQALGIEQNNVSLALSCIQAQMWVQSVIAGILRDGDNGILPTEIRK
Zebra finch   PLNSSLSSLGMGQWLVSEVLPTWEQISEKDHQVLLRALGMEQSNVSLALSCIQAQMWVQSVVAGILRDGDNGVLPTEIRK
GgLTR11       PLQSSLSALGTNQRFLSNVLSQWERTNIKDHQLIVDALAITQNNVSLALSC---------------/EGKGNALPTEIRK

              552                                                                           631
Chicken       IVWDAATEKERQLQAWWRLVNFTHDQVLNSVIAHVLTVVEARIEKVYPIVALGINTNGSVVYPLDHRMWARVSDRKWQSV
Zebra finch   IVWDAATEKERQLQAWWRLVNFTHDQALNAVVAHVLTVAEARIEKVYPIVALGVNTNGSVVYPLDHRMWARVSEGRWETV
GgLTR11       VIWDNANKFEREFQS*WHLVNFTYDPVNNXATAFVLTIRNASVYTIYPIIALGLNHNGVILYPLEXRVWAQQNGNKWQTV

              632                                                                           710
Chicken       DLEACILEQGLGFICEDDALKASDVCFDTSEGVCHFEINPRSNNKTMLVYVGKGCVCFRTMCKYVQINDIYNQT-VFNDS
Zebra finch   DLDACILERGLGFICEDDALKASDVCFDTKEGVCHFEINPQSSNKTMLVYVGKGCVCFRTACKYVQINEAYNQT-VFSDS
GgLTR11       DVSSCAVWEQQGFIC/ESNTIKAQDICLDTEQSICRFXRYPNETPEIVLIYIGKGCVCMRTSCNFILIGNITVDT--SNHS
TERV                       …VCESNALKA*DICLDIE N/VCHF*GTLQQVPETMLVYIGKGYVCIRIFVVSYS*IVLLELSAITLIF

              711                                                                           790
Chicken       NTCACNVAIIRGCDFVYKPPVFTSQLLIRNYSLYRSITPTPIGMDLSLVKEMLEHANLQQLLENAKAEAKKILITVHHDG
Zebra finch   NMCACNVATIRGCDFVYKPPVFTSQLLIRNYTLYRSITPTPIGMDLSLVKEMLEHANLQQLLENAKAEAKKILITVHHDG
GgLTR11       NVCVCNFTNIMGCDFNYSVPVTSYQLLWSNYTLIQDLPPAPIGMNLALVKKLLQHDDLYQLLERIRDNGK/KPLITVHHDT
TERV          NLSVYNFTSIVGCGFNY*VSVTAHQFLQADYTVIKDLSPVPSGMNLAFIRKLLQHDDLHQSLKHIRDNGQKTLITIHHDM
MMLV                                                       …LQNRRGLDLLFLKEGGLCAALKEECCFYADHTGLVRDSM
PERV                                                       …LQNRRGLDLLFLREGGLCAALKEECCFYVDHSGAIRDSM
                                                                   ISU

              791                                                                         865
Chicken       NVIKQIVERIKR--VGEHHWWEIFFGWSPTATGIFNALLHPVVIILL-MQMCVCFAMVATCYWIRQVRLCIENQLKGL…
Zebra finch   KVIKQVMERIKR--AGEHHWWEAFFGWSPTATGIFNALLHPIVVILL-MQICVCFAMVATCYRMRQVKLCFDNQMKEA…
GgLTR11       KEMHHVLERVKK--DGEHHWWETLLGWSPTARGVFNSVLHPVVILLILTLM--CLLLVIILYVKVWQMLR*
TERV          EEIHRVLERVKK--DGEHHWWEVLFGWSPTATGIFNSVLHPVIVVLGLVLL--CLV--LTVIYKD-VALAETSVPT*
MMLV          AKLRERLNQRQKLFESTQGWFEGLFNRSPWFTTLISTIMGPLIVLLMILLFGPCILNRLVQFVKDRISVVQALVLTQQ…
PERV          SKLRERLERRRREREADQGWFEGWFNRSPWMTTLLSALTGPLVVLLLLLTVGPCLINRFVAFVRERVSAVQIMVLRQQ…
```

(**A**) Hydrophobicity profile of chicken Ovex1 ORF3 protein (according to Kyte and Doolittle). The hatched area corresponds to the putative transmembrane anchor domain (TM). RX(R/K)R potential cleavage sites are indicated by arrows. Regions related to the RepBase GGLTR11 consensus sequence are represented by dark bars. (**B**) Chicken and zebra finch Ovex1 ORF3 putative protein sequences are aligned with the corresponding translation products of GGLTR11 and TERV, and with reference viral envelope proteins. Abbreviations and database accession numbers are given in Materials and methods. Residues are shaded when identical to those of the chicken ORF3 protein. X stands for an unknown amino acid, asterisk for a stop codon and slash for a frameshift introduced to optimize the alignment. In MMLV and PERV sequences, the immunosupressive domain (ISU) is underlined, and residues involved in the disulfide bond printed in bold characters.
