# Supplementary material for: OVEX1, a novel chicken endogenous retrovirus with sex-specific and left-right asymmetrical expression in gonads
Source: Retrovirology. 2009 Jun 17;6:59. doi: 10.1186/1742-4690-6-59 (PMC2717909; doi:10.1186/1742-4690-6-59)
Supplement: Additional file 6 — Table S7 – Polymorphisms in chicken Ovex1 sequences. Polymorphisms observed in the Ovex1 sequence between the Red Jungle Fowl (genomic sequence galGal3) and the white Leghorn chicken strain (this study). [file 1742-4690-6-59-S6.pdf]

Table S7 - Polymorphisms in chicken *Ovex1* sequences

Nucleotide sequence difference			Protein sequence difference			
Nucleotide position	Red Jungle Fowl[a]	White Leghorn[b]	Red Jungle Fowl	White Leghorn	Amino acid position	
116	G	G/A	Non translated			
165	A	G	Non translated			
930	G	A/G	silent		187	Gag-Pol
987	T	C/T	silent		206	Gag-Pol
1125	C	A	silent		252	Gag-Pol
1173	C	T/C	silent		268	Gag-Pol
1198	C	A	H	N	277	Gag-Pol
1236	A	A/G	silent		289	Gag-Pol
1329	T	T/C	silent		320	Gag-Pol
1344	C	G	silent		325	Gag-Pol
1566	G	A	silent		399	Gag-Pol
2046	A	G	silent		559	Gag-Pol
2427	C	C/T	silent		686	Gag-Pol
2523	C	C/G	silent		718	Gag-Pol
2532	G	T	silent		721	Gag-Pol
2853	A	A/C	silent		828	Gag-Pol
4092	T	G	silent		1241	Gag-Pol
4347	G	A	silent		1326	Gag-Pol
5307	T	C	silent		1646	Gag-Pol
5322	A	A/C	silent		1651	Gag-Pol
5532	A	G	I	M	1721	Gag-Pol
5574	A	G	silent		1735	Gag-Pol
5905	A	G	silent		43	ORF3
5972	A	G/A	T	A/T	66	ORF3
5975	T	C/T	Y	H/Y	67	ORF3
6070	A	G/A	silent		98	ORF3
6326	C	T/C	silent		184	ORF3
6460	A	G/A	silent		228	ORF3
6679	A	G/A	silent		301	ORF3
6713	A	C/A	S	R/S	313	ORF3
6802	T	C	silent		342	ORF3
7010	A	G/A	T	A/T	412	ORF3
7062	G	A/G	R	K/R	429	ORF3
7759	T	C	silent		661	ORF3
8162	A	G/A	I	V/I	796	ORF3
8251	G	A/G	silent		825	ORF3
8519	A	C	Non translated			

[a] *Gallus gallus* genome v2.1 (galGal3)
[b] This study
