# Supplementary material for: OVEX1, a novel chicken endogenous retrovirus with sex-specific and left-right asymmetrical expression in gonads
Source: Retrovirology. 2009 Jun 17;6:59. doi: 10.1186/1742-4690-6-59 (PMC2717909; doi:10.1186/1742-4690-6-59)
Supplement: Additional file 7 — Figure S8. Alignment of Ovex1 RT and homologous sequences. [file 1742-4690-6-59-S7.pdf]

Figure S8 – Alignment of Ovex1 RT and homologous sequences

CLUSTAL 2.0.3 multiple sequence alignment of RT domains

```
                                                        Domain 1
ALV                 PVWIDQWPLPEGKLVALTQLVEKELQLGHIEPS--LSCWNTPVFVIRKA-SG-----SYR 52
EAV-HP              PVWVDQWPLPKEKLDALQTLVARELRLGHIEPS--LSRWNTPVFVIQKK-SG-----AFR 52
MMTV                PVWLNQWPLKQEKLQALQQLVTEQLQLGHLEES--NSPWNTPVFVIKKK-SG-----KWR 52
HIV1                GPKVKQWPLTEEKIKALVEICTEMEKEGKISKIGPENPYNTPVFAIKKKDST-----KWR 55
HTLV1               PPEISQFPLNPERLQALQHLVRKALEAGHIEPY--TGPGNNPVFPVKKA-NG-----TWR 52
Xen1                LPRKPQYPLKPAQSESINKQLQTLLENGAIKRQ--SSPCNTPLFPVKEKGKAGEP-DKYR 57
DevI                YPRLPQYPLKAAQEQSLGVQITSLLRSGVLIRC--VSPCNTPLFPVKKKTAPGDP-PKYR 57
WEHV1               CPSIRQYPLPKEKVEGLRPMIHSLLAQGVLTEC--HSSCNTPIFPIKKPGR-----EEYR 53
WDSV                LPSIRQYPLPKDKTEGLRPLISSLENQGILIKC--HSPCNTPIFPIKKAGR-----DEYR 53
MMLV                PVSIKQYPMSQEARLGIKPHIQRLLDQGILVPC--QSPWNTPLLPVKKPGTN-----DYR 53
REV-A               PVRVRQYPITLEAKRSLRETIRKFRAAGILRPV--HSPWNTPLLPVRKSGTS-----EYR 53
GGLTR11             AVKQRQYPIPLERIMGLKPVIQTLVKDGLLELC--MSPYNTPILPVQKA-DG-----TYX 51
HERV-H              YPAQCQYPIPQHALKGLKPVITRLLQHGLLEPI--NSLFNSPILPVQKP-DK-----SYR 52
ZFERV               IVNVRQYKLRPEAVEGIGETIKELEAAEVLRRT--VSGWNTPILPLKKT-TG-----KYR 52
Chicken Ovex1       PPPQQQPQYPIEAEEGLWDTVKTLLDQGVLVEQ--QSTSNAMVWPLRKADRKT-----WR 53
Turkey Ovex1                …PVEAEEGLWDTIKTLLDQGVLVEQ--QSTSNAMLWPLRKADRKT-----WR 44
Guinea fowl Ovex1           …PVEAEEGLWDTVKTLLDQGVLVEQ--QSTSNALVWPLRKADKKT-----WR 44
Duck Ovex1                  …PAEAEEGLWDTVKTLLEQGVLVEQ--QSTSNAPVWPLRKADGKT-----WR 44
Zebra finch Ovex1   PPPQAQPWYPAEAEDSLWDTVKTLLDQGMLVEQ--QSTNNAPVWPLRKADGKM-----WK 53
SpeV                PPPQMQYKYPAETEKGIQAMIDSLLRQGVVVKM--QSVCNSPIWPVIKADEG------?D 50
FFV                 PTPQKQYHINPKAKPDIQIVINDLLKQGVLIQK--ESTMNTPVYPVPKPNGR------WR 52
HFV                 PRPQKQYPINPKAKPSIQIVIDDLLKQGVLTPQ--NSTMNTPVYPVPKPDGR------WR 52
MuERV-L             IVNQKQYCIP-GEIAEITATIKDLKDAGVVVPT--TSPFNSPIWPVQKTDGS------WR 51
HERV-L              ??IQKHYHHP-GGIAEISATIKDLKNAGVVIPI--TSLFNSPFWPVQKTDGS------WR 51
cENS3               LTCVKPYPLPLGARSGISPVLAELKEQGIVIPT--HSPFNSPVWPVRKPNGK------WR 52
RV_Tinamou          VTNVPQHPISAAARNGIAEVIADLGKEXMISRS--CSPC*STVWPVRKPDGR------WR 51
GGERV-L             TVHRRQYRTNRDSLLPIHKLIRQLESQGVISKT--HSPFNSPIWPVRKASGE------WR 52
SnRV                PKMIKQYPVPDASHASIKETVEALLEQGVLRKC--NSTVNSAIWPVGKPDGS------WR 52
```

```
                          Domain 2                Domain 3
ALV                 LLHDLRAVNAKLVPFGAVQQGAPVLSA--LPRGW-PLMVLDLKDCFFSIPLAEQDREAFA 109
EAV-HP              LLHDLRAVNSQLIPFGVVQQGAPVLSA--VPEEW-EVTAIDLKDCFFSIPLAEQDREAFA 109
MMTV                LLQDLRAVNATMHDMGALQPGLPSPVA--VPKGW-EIIIIDLQDCFFNIKLHPEDCKRFA 109
HIV1                KLVDFRELNKRTQDFWEVQLGIPHPAG--LKKKK-SVTVLDVGDAYFSVPLDEDFRKYTA 112
HTLV1               FIHDLRATNSLTIDLSSSSPGPPDLSS--LPTTLAHLQTIDLKDAFFQIPLPKQFQPYFA 110
Xen1                LVQDLRAVNEATVMETPLVSNPHTILSGIPP-SATHFRAVDLTNAFYSIPLREDCQYLFA 116
DevI                LVQDLRAVNSATILETPVVPNPNTLLSQVPT-SATLFTVIDLANAFFSVPLHEDSQFLFA 116
WEHV1               MIHDLRAINEIVAPLTAVVASPTTVLANLSP-DMTCFTVIDLSNAFFSVPIHPDSQYLFA 112
WDSV                MIHDLRAINNIVAPLTAVVASPTTVLSNLAP-SLHWFTVIDLSNAFFSVPIHKDSQYLFA 112
MMLV                PVQDLREVNKRVEDIHPTVPNPYNLLSGLPP-SHQWYTVLDLKDAFFCLRLHPTSQPLFA 112
REV-A               MVQDLREVNKRVETIHPTVPNPYTLLSLLPP-DRIWYSVLDLKDAFFCIPLAPESQLIFA 112
GGLTR11             LVQDLRKINEIVLKRHPLVPNPYTLMSXIPX-EHKWFSVIDLKDAFWTCPLDSESRDLFA 110
HERV-H              LVQNLRLINQIVLPIHPVVPNLYTLLSSIPS-STTHYSVLDLKDGFFTILLHSSSQPLFA 111
ZFERV               MVHDLRLINEKVLTATLPTPNPYTIMSKLTP-KHSHFTCIDLANAFFCMPLAEQCQGIFA 111
Chicken Ovex1       LMVNYSILNQVTPLKASIVTKYPDVMEAIFR-GSEWFSVLSLTSMSFAIPLHPESWHKFA 112
Turkey Ovex1        LTVNYSTLNQVTPLKASTVAKYPDVMEAIFR-GSEWFSVLSLTSVSFAIPLHPESWHKFA 103
Guinea fowl Ovex1   LTVNYSTLNQVTPLKTSTVTKYPDVMEAIFR-GSEWFSVLSLTSTSFAIPLHPESWHKFA 103
Duck Ovex1          LTVNYSALNQVTPLRTAVVAKYPNVMAAISR-GSKWFSVLSLTSASFAIPLHPESWHKFA 103
Zebra finch Ovex1   LTVDFSALNQVTPMQTPTVVRYPNIMAAISR-GSEWFSVLSLTSPSFAVPLHPDSWHKFA 112
SpeV                LPDDCRLL?????PFAPVVAKYNEIVAAIXP-WGPAGTVIDLANAFFAIPLYPACWYKFA 105
FFV                 MVLDYRAVNKVTPLIAVQNQHSYGILGSLFK--GRYKTTIDLSNGFWAHPIVPEDYWITA 110
HFV                 MVLDYREVNKTIPLTAAQNQHSAGILATIVR--QKYKTTLDLANGFWAHPITPESYWLTA 110
MuERV-L             MTVDYRKLNQVVTPIAAAVPDVVSLLEQINTSPGTWYAAIDLANAFFSVPVHKDHQKQIA 111
HERV-L              MRVDYHKLNQVVTPIAAAIQDVVSLLKQINTSPGTLYAAIDLANAFY--PFHKPHQKQFA 109
cENS3               LTIDYRRLNANTGPLTAAVPNISELIAAIQEQAHPFMATIDVKDMFFMVPLHPDDQLRFA 112
RV_Tinamou          LTIDYRRLNANTVPLTAAVPNIANLTATLAA-AHPWRATLDVKDVFFMAPLQEKDREKFT 110
GGERV-L             LTVDYRGLNEVTPPLSAAVPDMLELQYELESKAAKWYATTDIANAFFSIPLATECRPQFA 112
SnRV                LTIDYRPLNSAVSCPYPTVASTPELFAKLEK-KYQVYSSLDISNGFWSIRLEEECQYLFA 111
                                                        ↑
```

```
                                         Domain 4                  Domain 5

ALV                  FTLPSVNNQAPARRFQWKVLPQGMTCSPTICQLVVG-QVLEPLRLKHPS--LRMLHYMDD 166
EAV-HP               FTVPVSNNQRPTQRYQWRVLPQGMACSPTICQMVVG-KILGPLHHTSEASECIILHYMDD 168
MMTV                 FSVPSPNFKRPYQRFQWKVLPQGMKNSPTLCQKFVD-KAILTVRDKYQD--SYIVHYMDD 166
HIV1                 FTIPSINNETPGIRYQYNVLPQGWKGSPAIFQSSMT-KILEPFRKQNPD--IVIYQYMDD 169
HTLV1                FTVPQQCNYGPGTRYAWRVLPQGFKNSPTLFEMQLA-HILQPIRQAFPQ--CTILQYMDD 167
Xen1                 FTHE-------RQQYVWTVLPQGAQNSPTHFSLA---LTSILDSWISSHPEITLLQYVDD 166
DevI                 FTFQ-------GSQLTWTRTPQGAQNSPNQFTHA---MKMTLDPWVLQNLHVTLLQYVDD 166
WEHV1                FTFE-------GRQYTWTVLPQGFIHSPTLFSQA---LFSSLSKIKDSLTSEICI-YMDD 161
WDSV                 FTFE-------GHQYTWTVLPQGFIHSPTLFSQA---LYQSLHKIKFKISSEICI-YMDD 161
MMLV                 FEWRDPEM-GISGQLTWTRLPQGFKNSPTLFDEA---LHRDLADFRIQHPDLILLQYVDD 168
REV-A                FEWADAEE-GESGQLTWTRLPQGFKNSPTLFDEA---LNRDLQGFRLDHPSVSLLQYVDD 168
GGLTR11              FEWEDPET-GRK*QYRWIVLPQGFTESPNLFGQV---LEKVLEKFQVEEG-VKLLQYVDD 164
HERV-H               FTWTDPDT-HQSQQLTWTVLPQGFRDSPRYFSQA---LSHDLLSFHPSA--SHLIQYIDE 165
ZFERV                FSYQ-------GAQYTYNRLPQGFILSPGLFNQA---LRELLDSCTLHEG-TIVIQYVDD 160
Chicken Ovex1        FTLQ-------GRQFTFTRLPPGFHSTPTICHTH----VLKMWEKLS--HKESVLSCAGD 159
Turkey Ovex1         FTLQ-------GRQFTFTRLPPGFHSTPAICHTH----VLK…                   133
Guinea fowl Ovex1    FTLQ-------GRQFTFTRLPPGFHSTPAICHAH----VLK…                   133
Duck Ovex1           FTLR-------GRQFTFARVPPGFHNTPAICHMH----VTR…                   133
Zebra finch Ovex1    FTIR-------GRQFAFTRVPPGFHSAPAICHAR----VVRMWEQVS--HRESVLSCAGD 159
SpeV                 FTYR-------NQQYSFTRTPQGFHSSPSICHSV----VSKMWDKLKPESRGCVLSYVDD 154
FFV                  FTWQ-------GKQYCWTVLPQGFLNSPGLFTGD----VVDLLQGIPN-----VEVYVDD 154
HFV                  FTWQ-------GKQYCWTRLPQGFLNSPALFTAD----VVDLLKEIPN-----VQVYVDD 154
MuERV-L              FSWQ-------GQQYTFTVLPQVYINSPALCHNL----VRRDLDRLDLPQSITLVHYIDD 160
HERV-L               FSWQ-------GQQCIFTVLPQGYINSPALCHNL----IQSDLDHFSLPQDITLVHYIDD 158
cENS3                FTWE-------GQQYTFTRLPQGFKHSPTLAHYA----LAKELEQIPLEEGVRLYQYIDD 161
RV_Tinamou           FTWD-------GIQYTFNGLPQGYKHSPTIAHAA----LAGLLQKVSLPREVKLYQYVDD 159
GGERV-L              FTWR-------GVQYTWNRLPQGWKHSPTICHGL----IQTALEQGGAPEHL---QYIDD 158
SnRV                 FTFD-------TQQYTWTRLPQGFHASPGIFHQALYNGLASCKTAIESQGCK-LLQYVDD 163
                                                                           ↑↑
```

Alignment of chicken Ovex1 RT (residues 759 to 917), zebra finch RT deduced from the genomic sequence, partial RTs of turkey, guinea fowl and duck determined from the PCR-amplified fragments, and RTs of representative retroviral elements. Abbreviations and database accession numbers are given in Materials and methods. The alignment was performed with ClustalW2 using default settings and adjusted manually. Residues conserved in the five Ovex1 and SpeV sequences are underlined. Arrows show residues corresponding to the aspartate catalytic triad. RT domains are defined according to [58].
