# Supplementary material for: OVEX1, a novel chicken endogenous retrovirus with sex-specific and left-right asymmetrical expression in gonads
Source: Retrovirology. 2009 Jun 17;6:59. doi: 10.1186/1742-4690-6-59 (PMC2717909; doi:10.1186/1742-4690-6-59)
Supplement: Additional file 8 — Figure S9. Detection of apoptotic cells in chicken ovary at hatching. [file 1742-4690-6-59-S8.pdf]

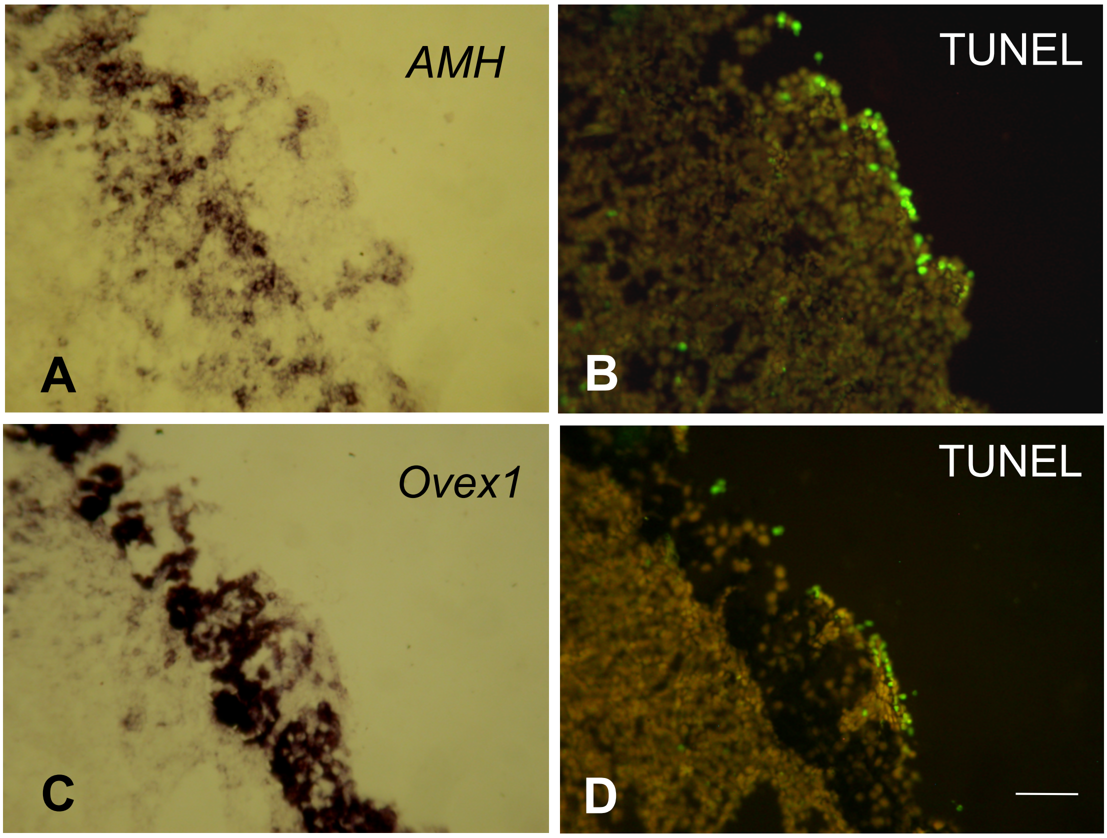

Figure S9 – Detection of apoptotic cells in the ovary at hatching

Double labeling for the detection of apoptotic cells by the TUNEL method (B and D) and of *AMH* (A) or *Ovex1* (C) expression by *in situ* hybridization of digoxygenin-labeled riboprobes on serial transverse cryostat sections of left ovary at hatching (P1). (A, B) and (C, D) correspond to double-labeled sections. Apoptotic cells are labeled in green. Scale bar = 100 µm.
