# Supplementary material for: OVEX1, a novel chicken endogenous retrovirus with sex-specific and left-right asymmetrical expression in gonads
Source: Retrovirology. 2009 Jun 17;6:59. doi: 10.1186/1742-4690-6-59 (PMC2717909; doi:10.1186/1742-4690-6-59)
Supplement: Additional file 9 — Table S1. Primers and PCR conditions. [file 1742-4690-6-59-S9.pdf]

Table S1. Primers and PCR conditions

Product	Forward primer [a]	Sequence[b]	Reverse primer [a]	Sequence[b]	PCR conditions
**RT-PCR fragment amplification**					
Fragment A	Ov31s	CCAGCAGAGCTCTGCAGCATCCATCC	Ov1171a	GGCTGACTGGCTCATCAGG	
Fragment B	Ov480s	ACTGTGTCAGCAGTGGGAGA	Ov3245a	GACACCTCTTTCCAGCACAG	
Fragment C	Ov2511s	TCCTGAGCTCCTCATCCATC	Ov5053a	ACCGTGAGAAGACATGCTCTA	
Fragment D	Ov4303s	AAACTGCAGTACCTCGCCAAG	Ov6849a	GGACGCAGGACAGCAATGATGGGA	57° 30 cycles
Fragment E	Ov5602s	TGGATGGGACCCGTTAGGGTTGTG	Ov7840a	GCACACACACCCTTTCCCTACGT	
Fragment F	Ov7594s	CACAAATGGGTCTGTGGTCT	Ov8904a	CACTGTCCTTGGCTTTCCCT	
**Race PCR**					
5' RACE			Ov6849a	GGACGCAGGACAGCAATGATGGGA	
5' RACE			Ov6061a	TACATGAGCTTGAGACCAAGGCCA	
5' RACE			Ov654a	GAGCTCACCCTTGGCCTGCTCCAGA	
3' RACE	Ov8378s	GCCAAGCGCCTCCTACCATAGTCA			
**Amplification of Gag and Pol DNA fragments from domestic birds**					
Gag	Ov480s	ACTGTGTCAGCAGTGGGAGA	Ov654a	GAGCTCACCCTTGGCCTGCTCCAGA	57° 30 cycles
			Ov656a	TTGAGCTCACCCTTGGCCTGCTC	59° 30 cycles
Pol	Ov2647s	CCTCCTCAGCAGCAGCCTCAATA	Ov3092a	TTGTGGCTGAGCTTCTCCCACAT	60° 30 cycles
**Expression**					
Unspliced	Ov31s	CCAGCAGAGCTCTGCAGCATCCATCC	Ov654a	GAGCTCACCCTTGGCCTGCTCCAGA	57° 27 cycles
Spliced	Episs	CGTCCGGACGAGGATTGATG[c]	Ov6369a	ACAAAGACTGGCTGGACTAATG	57° 29 cycles
GAPDH	GAPDHs	CACTATAAAGGCGAGATG	GAPDHa	GGCTGTGTGCCTGGCTCA	57° 26 cycles
**Internal polyadenylation site efficiency**					
	Ov5602s	TGGATGGGACCCGTTAGGGTTGTG	Ov6018a	TACCGCCTGACCCTGTGCTG	
			Ov6061a	TACATGAGCTTGAGACCAAGGCCA	57° 30 cycles
	Episs	CGTCCGGACGAGGATTGATG[c]	Ov6369a	ACAAAGACTGGCTGGACTAATG	
			Ov6849a	GGACGCAGGACAGCAATGATGGGA	

[a] Forward (s) and reverse primers (a) are named according to the position of their 5'-end in the DNA sequence given in Figs. 2 and 3.

[b] Sequences are written in the 5' to 3' direction

[c] Exon 1 is underlined and exon 2 doubly underlined.
